# V1 interneurons regulate the pattern and frequency of locomotor-like activity in the neonatal mouse spinal cord

**DOI:** 10.1371/journal.pbio.3000447

**Published:** 2019-09-12

**Authors:** Melanie Falgairolle, Michael J. O’Donovan

**Affiliations:** Developmental Neurobiology Section, National Institute of Neurological Disorders and Stroke, National Institutes of Health, Bethesda, Maryland, United States of America; NYU, UNITED STATES

## Abstract

In the mouse spinal cord, V1 interneurons are a heterogeneous population of inhibitory spinal interneurons that have been implicated in regulating the frequency of the locomotor rhythm and in organizing flexor and extensor alternation. By introducing archaerhodopsin into *engrailed-1*-positive neurons, we demonstrate that the function of V1 neurons in locomotor-like activity is more complex than previously thought. In the whole cord, V1 hyperpolarization increased the rhythmic synaptic drive to flexor and extensor motoneurons, increased the spiking in each cycle, and slowed the locomotor-like rhythm. In the hemicord, V1 hyperpolarization accelerated the rhythm after an initial period of tonic activity, implying that a subset of V1 neurons are active in the hemicord, which was confirmed by calcium imaging. Hyperpolarizing V1 neurons resulted in an equalization of the duty cycle in flexor and extensors from an asymmetrical pattern in control recordings in which the extensor bursts were longer than the flexor bursts. Our results suggest that V1 interneurons are composed of several subsets with different functional roles. Furthermore, during V1 hyperpolarization, the default state of the locomotor central pattern generator (CPG) is symmetrical, with antagonist motoneurons each firing with an approximately 50% duty cycle. We hypothesize that one function of the V1 population is to set the burst durations of muscles to be appropriate to their biomechanical function and to adapt to the environmental demands, such as changes in locomotor speed.

## Abstract 2

An optogenetic study in mice shows that inhibitory neurons that express engrailed-1 regulate the pattern and frequency of locomotor-like activity in the developing mouse spinal cord.

## Introduction

The analysis of the spinal circuitry responsible for orchestrating locomotion has been greatly facilitated by genetic strategies for identifying and manipulating different neuronal classes (for review, see [1]). One of the cell classes that has received considerable study is the V1 class of interneuron, which is composed of ipsilaterally projecting inhibitory interneurons that express the transcription factor *engrailed-1* (*En1*) [2–11]. The V1 group is heterogeneous, expressing a combination of 19 transcription factors [3], and contains an estimated 50 subclasses [5] including Renshaw cells and a subset of Ia inhibitory interneurons [2]. Using acute and chronic deletion strategies, V1 interneurons have been shown to regulate the locomotor frequency and flexor–extensor alternation, the latter in the absence of V2b inhibitory interneurons [6,12]. In the original paper describing the effects of V1 ablation or inhibition on locomotor activity, only one speed was investigated [6]. In subsequent work in the adult animal [4], V1 ablation changed the behavior of flexor and extensor bursts during different walking speeds. In control animals, as walking speed increases, the extensor burst durations decline, with little change in the duration of the flexor bursts. When V1 neurons were ablated, the speed independence of the flexor burst was abolished, and the flexor burst durations also declined with increasing walking speeds. This effect was not observed with deletion of V2b neurons, suggesting that the V1 population may be involved in adjusting the behavior of the flexor motoneuron population according to the prevailing biophysical demands. Supporting this hypothesis, it was recently shown that the V1 inputs vary according to the motor pool they target (hip, ankle, or foot), and this also applied to limb versus epaxial or hypaxial muscles [3,10]. Furthermore, in the tadpole and the zebrafish, it has been shown that interneurons expressing *En1* provide inhibition to limit the firing of motoneurons and interneurons during swimming and gate inhibition to sensory pathways [7,9]. In addition, it was shown recently that there are at least two types of V1 interneuron (slow and fast) in the zebrafish, which are selectively activated to regulate slow and fast swimming [8].

Although some acute (timescale of seconds) perturbations of V1 function have been reported, either using the allatostatin system [6] or optogenetics [4], most of the work examining the V1 population has been done in animals in which V1 neurons have been deleted or inactivated chronically [4,6,8,11], raising the possibility that compensatory mechanisms may complicate the interpretation of some of the earlier results. To circumvent this concern, we have used optogenetics to revisit the role of V1 interneurons in locomotor-like activity induced by drugs or by stimulation of dorsal or ventral roots in neonatal mice. For this purpose, we expressed the inhibitory opsin archaerhodopsin-3 (Arch) in *En1*-expressing neurons. The new experiments show that the function of the V1 interneuronal population is more complex than suggested by the earlier studies and reveal the existence of subsets of V1 interneurons that have specialized functional roles. For example, we find that acute hyperpolarization of V1 interneurons in the hemisected cord accelerates the locomotor rhythm in contrast to the slowing that occurs in the intact cord. In addition, V1 hyperpolarization produces a convergence of the duty cycles of flexor and extensor bursts toward 50%, suggesting that these neurons regulate asymmetrical activation of motor pools depending on their function or the environmental demands. Finally, these experiments allowed us to establish whether a subset of the V1 population—Renshaw cells—mediates the activation of the central pattern generator (CPG) by ventral root stimulation [13].

Some of this work has been presented in preliminary form [14].

## Results

### Expression of Arch in V1 interneurons

**Fig 1:**
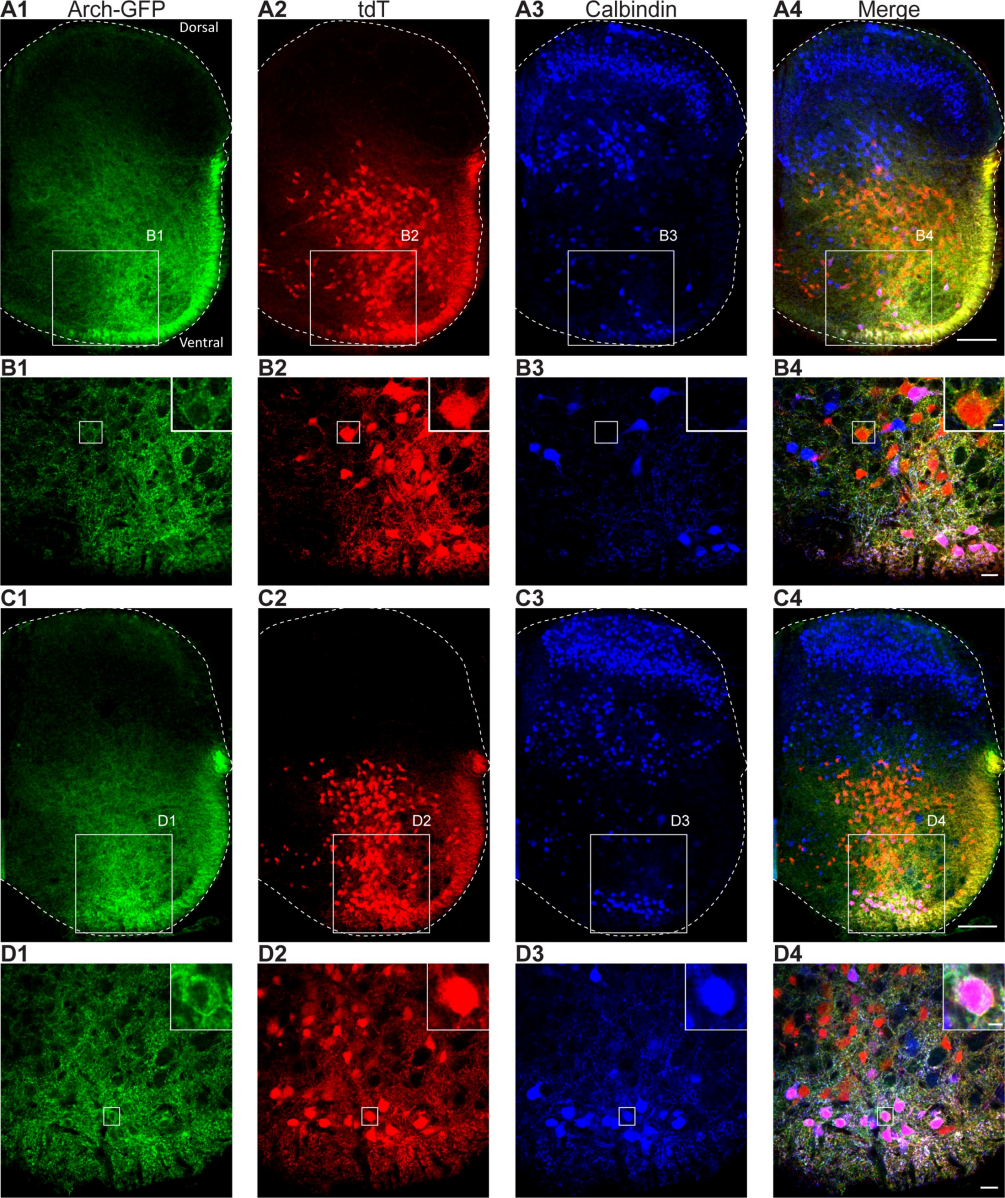
In *En1*-Arch-GFP-tdT cords, Arch-GFP is expressed in tdT-positive neurons and in a subset of ventrally located tdT-positive, calbindin-positive Renshaw cells. (A) Z-stack projection of low-magnification (10× objective) images (6.4 μm per optical section) of a 60-μm transverse section of the L2 segment from a P1 *En1*-Arch-GFP-tdT mouse spinal cord showing Arch-GFP expression (green, A1 and A4), tdT expression (red, A2 and A4), and calbindin-positive neurons (blue, A3 and A4) and the merged image (A4). The white scale bars represent 100 μm. (B) Single optical images (1 μm) acquired at a higher magnification (40× objective) from the region delineated by the white rectangle in (A). Insets at the top-right corner of the image show a magnified view of a single cell (white rectangle in the image) to show coexpression of tdT and Arch-GFP. The white scale bar is 20 μm, and 5 μm on the inset. (C) Z-stack projection of low-magnification (10× objective) images (6.4 μm per optical section) of a 60-μm transverse section of the L5 segment from a P1 *En1*-Arch-GFP-tdT mouse spinal cord showing Arch-GFP expression (green, C1 and C4), tdT expression (red, C2 and C4), and calbindin-positive neurons (blue, C3 and C4) and the merged image (C4). The white scale bars represent 100 μm. (D) Single optical images (1 μm) acquired at higher magnification (40× objective) from the location delineated by the white rectangle in (C). Insets at the top-right corner of the image show a magnified view of a single calbindin-positive cell (white rectangle in the image) to show coexpression of tdT and Arch-GFP. The white scale bar is 20 μm, and 5 μm on the inset. Arch, archaerhodopsin-3; *En1*, *engrailed-1*; GFP, green fluorescent protein; tdT, tdTomato.

To generate mice that express Arch bound to green fluorescent protein (GFP) (Arch-GFP) in *En1* interneurons, we used a genetic approach. Taking advantage of the cre-lox system, we bred mice expressing Cre in *En1* interneurons to mice with a cre-dependent Arch-GFP line that expresses in all tissues. In total, 50% of the offspring of this mating are expected to express Arch in V1 interneurons (*En1*^+/Cre^, Arch-GFP^+/flox^). As Arch-GFP is membrane bound, it is difficult to verify that it expresses in *En1*-positive neurons. To ensure that it does, in some animals we coexpressed tdTomato (tdT)—which fills the neuronal cytoplasm—together with Arch-GFP in *En1* interneurons. To do so, we first generated mice that express tdT in V1 interneurons (*En1*^+/Cre^, tdT^flox/flox^), and we crossed these mice to the cre-dependent Arch-GFP to generate the dual expression (*En1*^+/Cre^, tdT^+/flox^, Arch-GFP^+/flox^). As illustrated in Fig 1, Arch-GFP and tdT were densely expressed in neurons and their processes in the ventral half of the cord (*n* = 3), consistent with previous reports showing that V1 interneurons are located ventrally in the spinal cord [2–4,6,12] in both the L2 (Fig 1A and 1B) and L5 (Fig 1C and 1D) segments. Ventrally located neurons expressing calbindin and tdT (presumably Renshaw cells [2–4]) also expressed GFP (Fig 1D inset). As expected, other neurons that were not calbindin-positive also expressed both tdT and GFP (Fig 1B inset), consistent with previous studies [2]. We also verified that in preparations expressing only Arch-GFP in *En1* interneurons, the motoneurons were unlabeled (S1 Fig).

**Fig 2:**
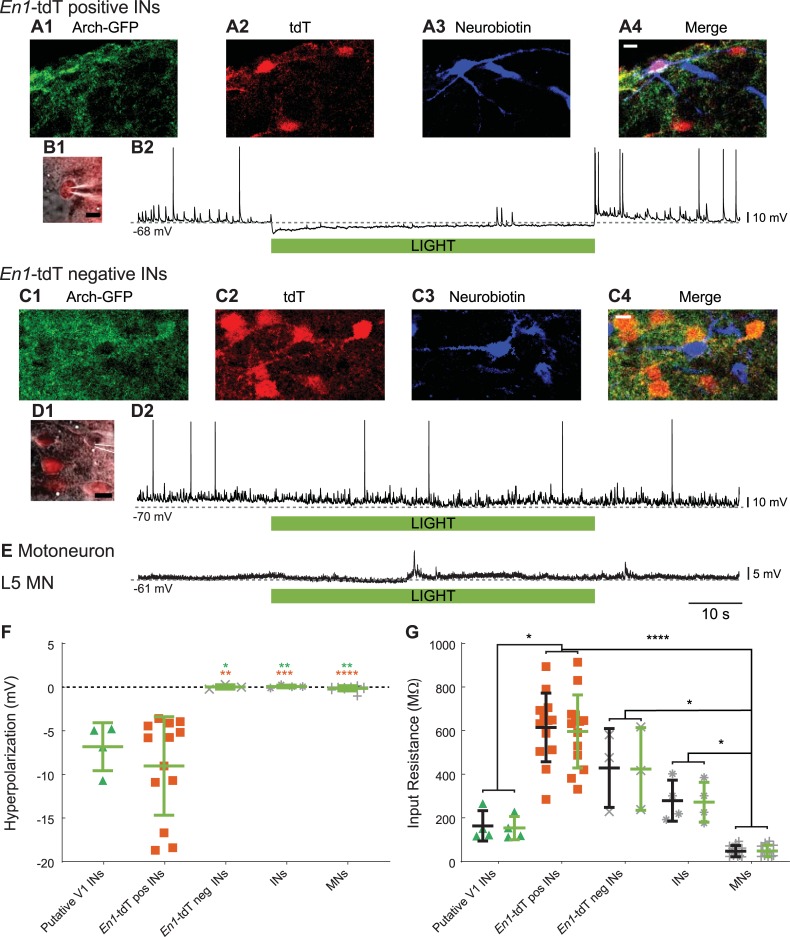
Whole-cell recordings showing the effects of light on V1 INs and MNs in *En1*-Arch-GFP and *En1*-Arch-GFP-tdT mouse spinal cords. (A) Single optical sections (2 μm) of a 100-μm-thick transverse section showing an IN intracellularly filled with Neurobiotin (blue, A3 and A4) expressing both Arch-GFP (green, A1 and A4) and tdT (red, A2 and A4). The white scale bar in A4 is 10 μm. (B1) High-contrast, bright-field image with tdT fluorescence superimposed showing the electrode and the visually targeted tdT-pos IN in a dorsally shaved (between the L2 and L5 ventral roots) *En1*-Arch-GFP-tdT mouse spinal cord. The black scale bar represents 10 μm. (B2) Intracellular recording of the IN in B1, showing hyperpolarization upon illumination. The duration of the light (60 s) is indicated by the green bar. (C) Single optical sections (2 μm) showing an intracellularly filled tdT-neg IN from a 100-μm-thick transverse section. Panels C1–C4 as in A1–A4. (D1) High-contrast, bright-field image with tdT fluorescence superimposed showing the electrode and a visually targeted tdT-neg IN. The black scale bar represents 10 μm. (D2) Intracellular recording of the IN in D1, showing no hyperpolarization upon illumination. The duration of the light (60 s) is indicated by the green bar. (E) Intracellular recording of a MN recorded in an *En1*-Arch spinal cord showing no hyperpolarization upon illumination. The duration of the light (60 s) is indicated by the green bar. (F) Plot showing the light-induced hyperpolarization under control conditions for all neurons recorded in this study. All neurons expressing tdT in *En1*-Arch-GFP-tdT spinal cords were hyperpolarized, whereas neurons neg for tdT were not. MNs did not show any hyperpolarization in *En1*-Arch-GFP preparations. Unidentified INs in *En1*-Arch-GFP preparations were classified as putative V1 INs or non-V1 INs according to their response to the light. Both putative V1 INs (green triangles) and *En1*-tdT-pos INs (red squares) showed a significant hyperpolarization when compared with the other neuronal groups (Kruskall-Wallis test *p* < 0.0001; putative V1 INs versus *En1*-tdT-pos INs *p* = 0.8921, putative V1 INs versus *En1*-tdT-neg INs *p* = 0.0224, putative V1 INs versus INs *p* = 0.0046, putative V1 INs versus MNs *p* = 0.091; *En1*-tdT-pos INs versus *En1*-tdT-neg INs *p* = 0.0045, *En1*-tdT-pos INs versus INs *p* = 0.0003, *En1*-tdT-pos INs versus MNs *p* < 0.0001; *En1*-tdT-neg INs versus INs *p* = 0.7325, *En1*-tdT-neg INs versus MNs *p* = 0.7131; INs versus MNs *p* = 0.3881; green * represents the *p*-value comparing putative V1 INs and the other neuronal classes; red * is for the *p*-value comparing *En1*-tdT-pos cells and the other neuronal classes). (G) Input resistance of all the INs and MNs recorded in this study under control conditions (black crosses) and during illumination (green crosses), separated according to their putative identities. Putative V1 INs (green triangles; *n* = 4 in 4 *En1*-Arch spinal cords, control versus light, Wilcoxon signed-rank test *p* = 0.375), *En1*-tdT-pos INs (red rectangles; *n* = 13 in four *En1*-Arch-tdT spinal cords, Wilcoxon signed-rank test *p* = 0.1272), *En1*-tdT-neg INs (*En1*-tdT-neg, gray Xs; *n* = 3 in one *En1*-Arch-tdT spinal cord, Wilcoxon signed-rank test *p* > 0.9999), putative non-V1 INs (gray stars; *n* = 4 in two *En1*-Arch spinal cords, Wilcoxon signed-rank test *p* = 0.5), and MNs (gray pluses; *n* = 12 in 12 *En1*-Arch spinal cords, Wilcoxon signed-rank test *p* = 0.0771). Input resistances were also compared among the groups before illumination (Kruskall-Wallis test, *p* < 0.0001; multiple comparison putative V1 INs versus *En1*-tdT-pos INs *p* = 0.0160; putative V1 INs versus *En1*-tdT-neg INs *p* = 0.3143; putative V1 INs versus INs *p* = 0.6182; putative V1 INs versus MNs *p* = 0.1292; *En1*-tdT-pos INs versus *En1*-tdT-neg INs *p* = 0.3418; *En1*-tdT-pos INs versus INs *p* = 0.073; *En1*-tdT-pos INs versus MNs *p* < 0.0001; *En1*-tdT-neg INs versus INs *p* = 0.5859; *En1*-tdT-pos INs versus MNs *p* = 0.0101; INs versus MNs *p* = 0.0334). *p < 0.05, **p < 0.01, ***p < 0.001, ****p < 0.0001. The data underlying this figure can be found in S1 Data. Arch, archaerhodopsin-3; *En1*, *engrailed-1*; GFP, green fluorescent protein; IN, interneuron; MN, motoneuron; neg, negative; pos, positive; tdT, tdTomato.

To confirm that V1 interneurons were hyperpolarized during illumination, 13 tdT-positive (Fig 2A and 2B) and three tdT-negative interneurons (Fig 2C and 2D) were visually patched in *En1*-tdT-Arch spinal cords. For this purpose, we removed the dorsal part of the spinal cord between the L2 and L5 ventral roots to provide visual access to the tdT-labeled neurons. We found that all the tdT-positive neurons were hyperpolarized by the light (Fig 2F, mean −9.0 ± 5.6 mV, range −3.6 to −18.7 mV, *n* = 13) and that none of the tdT-negative neurons were (Fig 2F, mean −0.03 ± 0.3 mV, range −0.22 to 0.29 mV, *n* = 3). Moreover, in *En1*-Arch cords, we recorded intracellularly from four ventrally located interneurons that were hyperpolarized during exposure to green light, consistent with them expressing Arch (Putative V1 IN, Fig 2F) with no statistically significant change in their input resistance (Fig 2G). Similar recordings from four additional interneurons and 12 antidromically identified motoneurons revealed no changes in either membrane potential (Fig 2E and 2F) or input resistance (Fig 2G) during illumination, suggesting that bystander effects [15] are minimal. Unless otherwise specified, experiments were performed in *En1*-Arch-GFP spinal cords (*En1*-Arch).

### The effects of hyperpolarizing *En1*-positive interneurons on fictive locomotion evoked by sacro-caudal afferent stimulation

**Fig 3:**
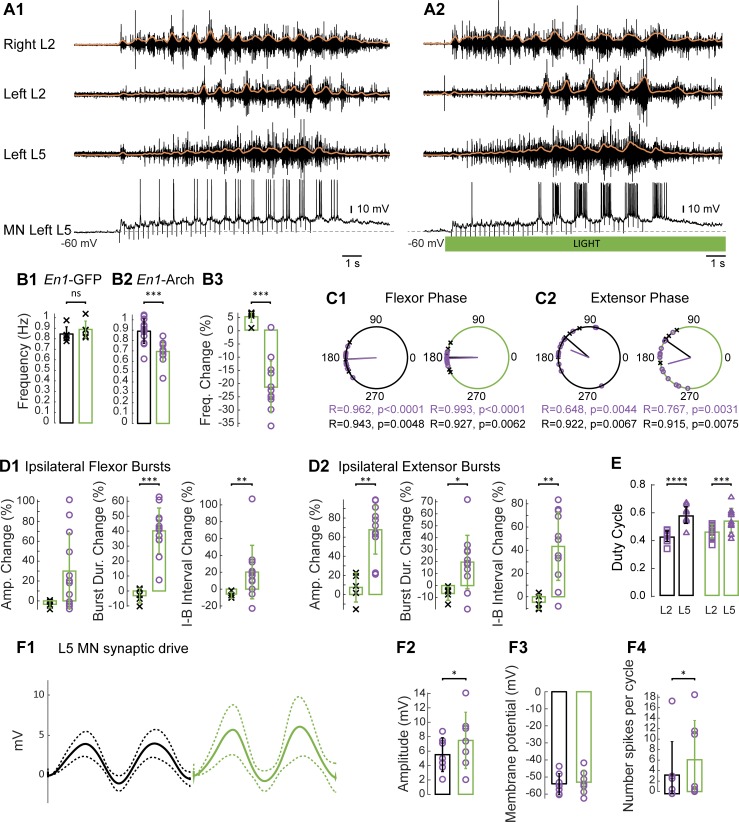
Hyperpolarization of V1 interneurons slows dorsal root–evoked fictive locomotion and enhances rhythmic drive. (A) Fictive locomotion evoked by sacral dorsal root stimulation before (A1) and during (A2) hyperpolarization of *En1*-positive neurons in an *En1*-Arch spinal cord. The black traces show the high-pass (10 Hz) filtered signal from the right and left L2 and the left L5 ventral roots together with an intracellular recording from an extensor MN in the left L5 segment. The superimposed orange traces are the integrated neurograms. The green bar indicates the duration of the light. (B) Average frequency of dorsal root–evoked locomotor-like activity without (black rectangle) and in the presence of green light (green rectangle) in *En1*-GFP (*n* = 5; black crosses, [B1]) and *En1*-Arch (*n* = 12; purple circles, [B2]) spinal cords (Wilcoxon signed-rank test, *p* = 0.0625, *p* = 0.001, respectively). (B3) Bar plot showing the change in frequency of *En1*-GFP (black crosses) and *En1*-Arch (purple circles) preparations during illumination compared with the frequency before illumination (Mann-Whitney test, *p* = 0.0006). (C) Circular plots showing the phasing of the bilateral flexor (C1) and the ipsilateral flexor–extensor (C2) ventral roots during dorsal root–evoked fictive locomotion without (black circles) or with (green circles) illumination of *En1*-GFP (black crosses) and *En1*-Arch (purple circles) cords. R is the length of the vector, and *p* is the value for the Rayleigh test of uniformity. Using the Harrison-Kanji test, we calculated the statistical differences between the two groups of animals (genetic identity *En1*-GFP versus *En1*-Arch) and the differences between light on and light off (light status) for the phasing in the bilateral L2 ventral roots. The results of the tests were light status: F(1, 30) *p* = 0.71, genetic identity: F(1, 30) *p* = 0.65, and interaction: F(1, 30) *p* = 0.80. The same test was performed for the flexor–extensor phases (light status: F[1, 30] *p* = 0.11, genetic identity: F[1, 30] *p* = 0.029, interaction: F[1, 30] *p* = 0.46). (D1) Characteristics of the L2 flexor-related ventral root bursts ipsilateral to the recorded extensor-related L5 bursts. Bar plot representing the percentage change during illumination of the amplitude (left panel), the Burst Dur. (middle panel), and the I-B (right panel) in *En1*-GFP (black crosses) and *En1*-Arch (purple circles) preparations (Mann-Whitney test, *p* = 0.0637, *p* = 0.0003, *p* = 0.0094, respectively) compared with the prelight values. (D2) Characteristics of the L5 extensor-related ventral root bursts ipsilateral to the recorded L2 flexor-related L5 bursts. Bar plot representing the percentage change during illumination of the amplitude (left panel), the Burst Dur. (middle panel), and the I-B interval (right panel) in *En1*-GFP (black crosses) and *En1*-Arch (purple circles) preparations (Mann-Whitney test, *p* = 0.0013, *p* = 0.0194, *p* = 0.0023, respectively) compared with the prelight values. (E) Duty cycle of the L2 (purple square) and L5 (purple triangles) cycles in *En1*-Arch cords before (black rectangles) and during (green rectangles) illumination (two-way RM ANOVA, light status: F[1, 11] *p* = 0.9653, roots: F[1, 11] *p* = 0.0003, interaction: F[1, 11] *p* = 0.0114). (F1) Cycle-triggered average (continuous line) and standard deviation (dotted lines) of the synaptic drive to MNs during dorsal root–evoked fictive locomotion (two cycles) in an *En1*-Arch spinal cord, without (black) and with (green) illumination. Bar plot of the amplitude of the synaptic drive (F2), the membrane potential at the trough of the cycle (F3), and the number of spikes per cycle (F4) in MNs (*n* = 7) recorded in *En1*-Arch spinal cords during dorsal root–evoked locomotor-like activity without (black bars) or with (green light) illumination (Wilcoxon test, *p* = 0.0313, *p* = 0.4688, *p* = 0.0313, respectively). *p < 0.05, **p < 0.01, ***p < 0.001, ****p < 0.0001. The data underlying this figure can be found in S2 Data. Amp. Change, amplitude change; Arch, archaerhodopsin-3; Burst Dur., burst duration; *En1*, *engrailed-1*; Freq. Change, frequency change; GFP, green fluorescent protein; I-B, interburst; MN, motoneuron; ns, not significant; RM, repeated measures.

To establish the effects of hyperpolarizing the V1 population on locomotor-like activity, we first examined the effects of light on fictive locomotion evoked by sacral dorsal root stimulation [16,17]. Fig 3A shows locomotor-like activity evoked by sacral afferent stimulation in an *En1*-Arch preparation under control conditions (no light) followed by the same stimulation while illuminating the lumbar cord with green light to hyperpolarize V1 interneurons (*n* = 12; Fig 3A2). In the *En1*-Arch cords, the light induced a 21.6% ± 10.23% reduction (Fig 3B3, purple circles) in the frequency of the locomotor rhythm from 0.89 (Fig 3B; black rectangle) to 0.69 Hz (Fig 3B2; green rectangle, Wilcoxon test, *p* = 0.001). In intracellularly recorded motoneurons (*n* = 7), light increased both the amplitude of the rhythmic synaptic drive (from 5.51 ± 2.35 to 7.48 ± 3.91 mV, Fig 3F1 and 3F2; Wilcoxon signed-rank test, *p* = 0.0313) and the number of spikes in each burst (from 3.55 ± 6.39 to 6.09 ± 7.48, Fig 3F4; Wilcoxon signed-rank test, *p* = 0.0313), whereas there was no change in the baseline membrane potential (from −54.01 ± 6.2 to −53.04 ± 6.9 mV, Fig 3F3; Wilcoxon signed-rank test, *p* = 0.4688). To assess whether the light itself produced nonspecific effects, we performed the same experiments on preparations expressing GFP instead of the opsin (S2 Fig) and quantified the effects on locomotor-like activity. We found that light induced a small but insignificant increase in the frequency of the rhythm in the *En1*-GFP cords (Fig 3B1; black crosses 5.06% ± 2.42%, from 0.85 [black rectangle] to 0.89 Hz [green rectangle], Wilcoxon signed-rank test, *p* = 0.0625), which was significantly different from the decrease in the frequency observed in the *En1*-Arch cords (Fig 3B3, 21.6% ± 10.23% Mann-Whitney test, *p* = 0.0006). We found no statistical difference in the phasing of ventral root activity in the *En1*-GFP and *En1*-Arch cords, with or without illumination (Fig 3C, black and green circles, respectively, Watson-Williams test, *p* > 0.05). We did observe a light-induced increase in the amplitude of the extensor-related ventral root bursts of 67.77% ± 25.49% (Fig 3A and 3D2 left panel, *p* = 0.0085), whereas the increase in the flexor-related ventral root burst 30.03% ± 37.88% was not statistically different from that observed in the *En1*-GFP cords (Fig 3D1 left panel, *p* = 0.0631). The pattern of rhythmic activity was also affected by the light in the *En1*-Arch cords. Specifically, there was an increase in the duration of the flexor-related and extensor-related bursts and the accompanying interburst intervals that was not observed in the *En1*-GFP cords (Fig 3D1 and 3D2 middle and right panels). These changes were not identical in the flexor-dominated and extensor-dominated roots, with the greatest increase in burst duration occurring in the flexor-dominated roots (the flexor burst duration increased by 40.2% ± 15.25% from 0.45 ± 0.1 s to 0.62 ± 0.14 s, whereas the extensor burst duration increased by 19.63% ± 22.37% from 0.61 ± 0.72 s to 0.72 ± 0.15 s, Wilcoxon signed-rank test, *p* = 0.0093). Because of the asymmetrical changes in these parameters in the flexor-dominated and extensor-dominated roots, there was a small change in the duty cycle of the two roots, with a convergence toward equality, when the light was turned on (Fig 3E).

In another set of experiments, we obtained whole-cell recordings from visually identified *En1*-tdT-Arch neurons (*n* = 4 cords) after removal of the dorsal part of the lumbar cord to gain access to the labeled V1 interneurons. In two of the cords, we were able to trigger rhythmic activity by stimulating sacro-caudal afferents. In the other two cords, the dorsal removals were deeper, and rhythmic activity could not be evoked. Five *En1*-tdT-positive neurons were patched in the two cords showing rhythmic behavior (four in the first and one in the second preparation). Under control conditions, none of the cells exhibited rhythmic synaptic drive (S3A1 and S3B1 Fig). However, all of them showed reduced spiking during illumination (S3A2 and S3B2 Fig). In two cells, rhythmic drive potentials appeared during illumination (S3A2 Fig), either because of enhanced rhythmic drive or because of the light-induced hyperpolarization.

### Hyperpolarizing V1 interneurons does not prevent ventral root–evoked fictive locomotion

**Fig 4:**
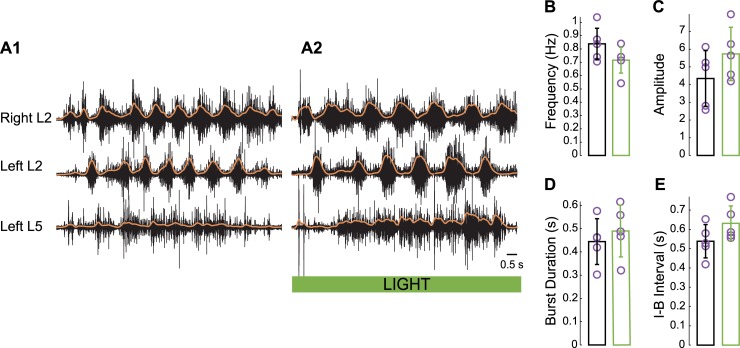
Hyperpolarization of V1 interneurons does not abrogate ventral root–evoked, locomotor-like activity. (A) Ventral root–evoked fictive locomotion before (A1) and during (A2) hyperpolarization of V1 interneurons. (B-E) Average frequency (B), amplitude (C), burst duration (D), and the I-B interval (E) of ventral root–evoked, locomotor-like activity measured in a flexor-related segment (L2) before and after hyperpolarization of V1 interneurons (Wilcoxon signed-rank test, *n* = 5, B: *p* = 0.125, C: *p* = 0.1875, D: *p* = 0.3125, E: *p* = 0.3125, respectively). The data underlying this figure can be found in S3 Data. I-B, interburst.

Locomotor-like activity can be evoked by stimulation of the ventral roots [13], which could be mediated by motoneuronal projections to Renshaw cells (a subset of V1 interneurons [2]) within the spinal cord. If this pathway is involved, hyperpolarizing V1 interneurons, including Renshaw cells, should block ventral root–evoked fictive locomotion. To test this idea, we examined the ability of ventral root stimulation (L5 or L6) to evoke locomotor-like activity during hyperpolarization of V1 neurons by light in *En1*-Arch cords. We found that hyperpolarizing V1 interneurons did not block the ability of the preparation to produce locomotor-like activity when stimulating the ventral root (Fig 4A). Furthermore, during illumination, the changes in locomotor-like activity were similar to those observed when the dorsal roots were stimulated (Fig 4A2). These included a decrease in the frequency, an increase in the burst amplitude, and a lengthening of the burst and interburst interval duration compared with the prelight values (Fig 4B, 4C, 4D and 4E, respectively), although the values were not statistically different from those before the light.

### Effect of light on locomotor-like activity evoked by drugs in *En1*-Arch cords

**Fig 5:**
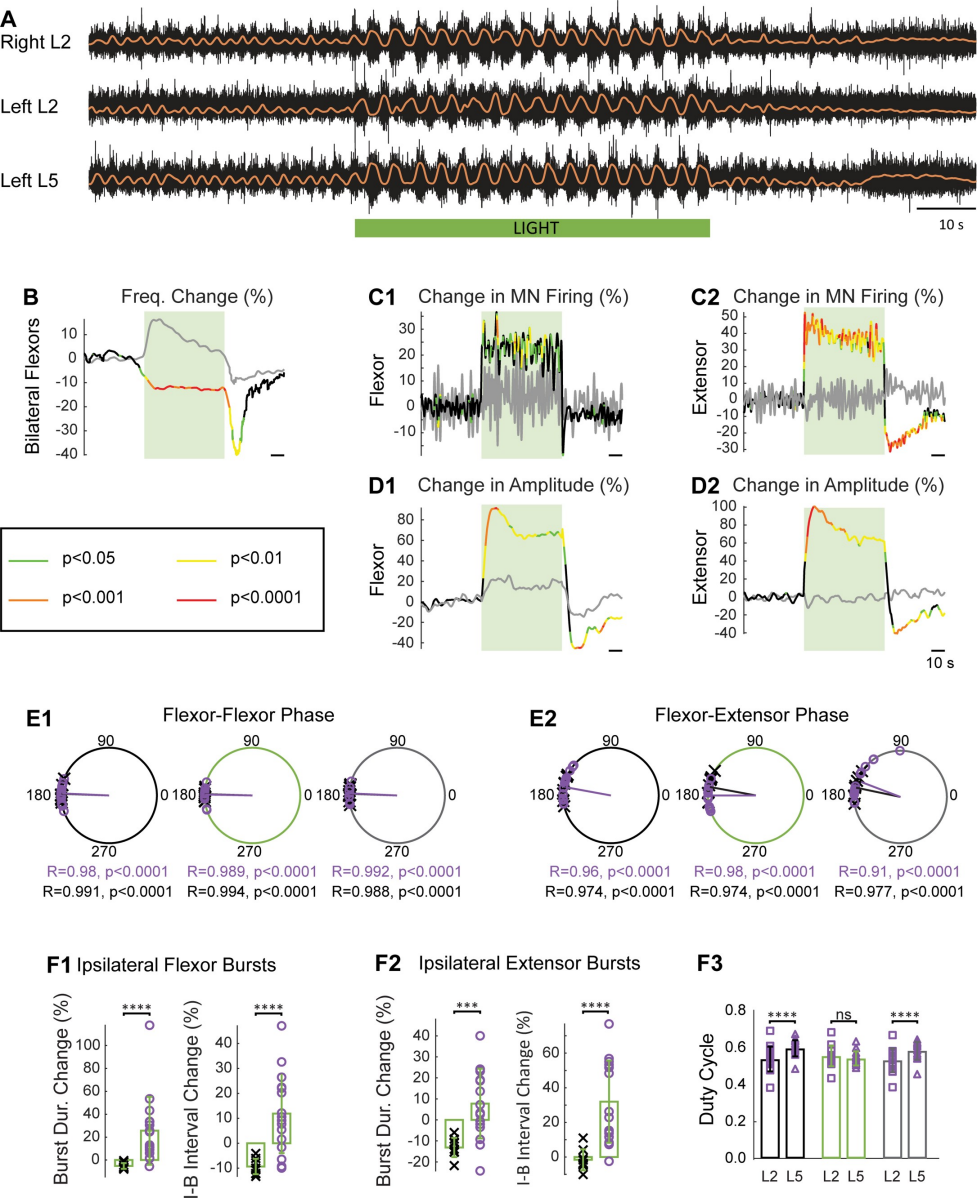
During drug-induced locomotor-like activity, light-induced hyperpolarization of V1 interneurons slows the rhythm and changes its pattern. (A) Locomotor-like activity evoked by 5 μM NMDA, 10 μM 5-HT, and 50 μM DA in an *En1*-Arch spinal cord. The black traces show the high-pass (10 Hz) filtered signal from the right and left L2 and the left L5 ventral roots. The superimposed orange traces are the integrated neurograms. The green bar indicates the duration of the light (60 s). (B) Time series showing the change in frequency averaged for all experiments for the bilateral flexor roots from *En1*-Arch preparations (black lines) and *En1*-GFP preparations (gray lines). (C) Change (%) in the averaged integrated ventral root discharge (Change in MN Firing) for the ipsilateral flexor (C1) and extensor (C2) ventral roots for *En1*-Arch preparations (black lines) versus *En1*-GFP preparations (gray lines). (D) Time series showing the change (%) in the averaged amplitude (trough to peak) of the integrated ventral root discharge (Change in Amplitude) for the ipsilateral flexor (D1) and extensor (D2) ventral roots for *En1*-Arch preparations (black lines) and *En1-*GFP preparations (gray lines). (B–D) The statistics were obtained using a bootstrap *t* test between *En1*-Arch (*n* = 16) and *En1*-GFP cords (*n* = 9), and *p*-values are color coded as indicated in the box in the left panel. (E) Circular plots showing the phasing of the bilateral flexor (E1) and the ipsilateral flexor–extensor (E2) ventral roots during fictive locomotion before (black circles), during (green circles), and after (gray circles) illumination of *En1*-GFP (black crosses) and *En1*-Arch (purple circles) cords. R is the length of the vector, and *p* is the value for the Rayleigh test of uniformity. Using the Harrison-Kanji test, we calculated the statistical differences between the two groups of animals (genetic Identity) and the differences before, during, and after illumination (light status) for the phasing in the bilateral L2 and ipsilateral L2–L5 ventral roots. The result of the test for bilateral L2 phase was light status: F(2, 69) *p* = 0.88, genetic identity: F(1, 69) *p* = 0.9, and interaction: F(2, 69) *p* = 0.80. The same test was performed for the flexor–extensor phases (light status: F[2, 69] *p* = 0.02, genetic identity: F[1, 69] *p* = 0.59, interaction: F[2, 69] *p* = 0.18). We then performed a Watson-Williams test to compare *En1*-Arch L5 phasing before, during, and after the light (*p* = 0.0138). (F1) Light-dependent changes in flexor-related ventral root bursts ipsilateral to the recorded extensor-related burst for *En1*-GFP and *En1*-Arch cords. Bar plot showing the light-induced change in the burst characteristics: duration of the burst (left panel) and the I-B interval (right panel) during (green rectangles) illumination compared with the durations before the light was turned on (% change) in *En1*-GFP (black crosses) and *En1*-Arch (purple circles) preparations. (F2) Similar measurements as (F1) for extensor-related ventral root bursts ipsilateral to the recorded flexor-related bursts. Two-way ANOVA was used to statistically compare the Burst Dur. between L2 and L5 in *En1*-GFP and *En1*-Arch (light status: F[2, 207] *p* < 0.0001, genetic identity: F[5, 207] *p* < 0.0001, interaction: F[10, 207] *p* < 0.0001) and the duration of the I-B interval (light status: F[2, 207] *p* < 0.0001, genetic identity: F[5, 207] *p* < 0.0001, interaction: F[10, 207] *p* < 0.0001). (F3) Duty cycle of the L2 (purple squares) and L5 (purple triangles) cycles in *En1*-Arch cords before (black rectangles), during (green rectangles), and after (gray rectangles) illumination (two-way RM ANOVA, light status: F[2, 30] *p* = 0.4866, roots: F[1, 15] *p* = 0.2642, interaction: F[2, 30] *p* < 0.0001). ***p < 0.001, ****p < 0.0001. The data underlying this figure can be found in S4 Data. 5-HT, 5-hydroxytryptamine; Arch, archaerhodopsin-3; Burst Dur., burst duration; DA, dopamine; *En1*, *engrailed-1*; Freq. Change, frequency change; GFP, green fluorescent protein; I-B, interburst; MN, motoneuron; NMDA, N-methyl-D-aspartate; ns, not significant; RM, repeated measures.

**Fig 6:**
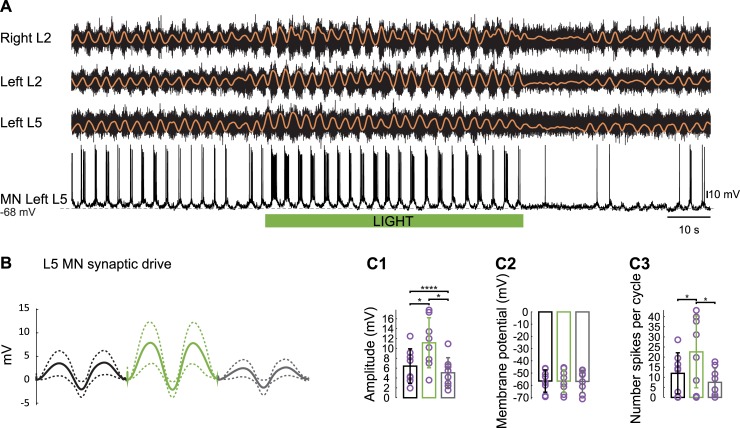
Light-induced hyperpolarization of V1 interneurons increases the rhythmic synaptic drive to MNs during drug-induced locomotor-like activity. (A) Locomotor-like activity evoked by 5 μM NMDA, 10 μM 5-HT, and 50 μM DA in an *En1*-Arch spinal cord. The black traces show the high-pass (10 Hz) filtered signal from the right and left L2 and the left L5 ventral roots together with a flexor MN recorded from the left L5 segment. The superimposed orange traces are the integrated neurograms. (B) Cycle-triggered average (continuous line) and standard deviation (dotted lines) of the synaptic drive to an MN during fictive locomotion (two cycles) in an *En1*-Arch spinal cord (*n* = 8) before (black), during (green), and after (gray) illumination. (C) Bar plot of the amplitude of the synaptic drive ([C1]; Friedman test, *p* < 0.0001), the membrane potential ([C2]; Friedman test, *p* > 0.9999), and the number of spikes per cycle ([C3]; Friedman test, *p* = 0.0072) in MNs recorded in an *En1*-Arch spinal cord during fictive locomotion before (black), during (green), and after (gray) illumination. *p < 0.05, ****p < 0.0001. The data underlying this figure can be found in S5 Data. 5-HT, 5-hydroxytryptamine; Arch, archaerhodopsin-3; DA, dopamine; *En1*, *engrailed-1*; MN, motoneuron; NMDA, N-methyl-D-aspartate.

In the next experiments, we investigated the effects of hyperpolarizing V1 interneurons during fictive locomotion produced by a drug cocktail (5 μM N-methyl-D-aspartate [NMDA], 10 μM 5-hydroxytryptamine [5-HT], and 50 μM dopamine [DA]). We found that hyperpolarizing V1 interneurons during fictive locomotion decreases the locomotor-like frequency (Fig 5A and 5B), as shown previously in experiments in which the V1 neurons were genetically deleted or pharmacologically hyperpolarized [6]. To ensure that the light-induced changes were due to the hyperpolarization of V1 interneurons and not to nonspecific effects of the light, we also examined the effects of light on drug-induced locomotor-like activity in *En1*-GFP spinal cords (S4 Fig) and compared these results statistically to those obtained in the *En1*-Arch animals using a bootstrap *t* test (see [18], Fig 5B, 5C, and 5D). Hyperpolarization of V1 interneurons in the *En1*-Arch cords decreased the locomotor-like frequency by approximately 15% (from 0.33 ± 0.06 Hz to 0.29 ± 0.06 Hz; Fig 5B, *p* < 0.0001), whereas illumination of the *En1*-GFP cords increased the frequency by approximately 15% at the peak of the increase (from 0.36 ± 0.06 Hz to 0.4 ± 0.07 Hz). In the *En1*-Arch cords, after the light was turned off, there was often a transient disruption of the rhythm, with a decrease in the amplitude or lack of bursting in the ventral root recordings and an absence of rhythmic drive potentials in motoneurons (Figs 5A and 6A). This was the reason for the sharp decrease in the frequency of the rhythm after the light was turned off (Fig 5B). In some of the *En1*-GFP cords, a small decrease in the frequency of the rhythm was also observed when the light was turned off (S4B Fig). In the *En1*-Arch cords, light increased motoneuron firing in both the flexor-dominated and extensor-dominated ventral roots, as manifested by the increased trough-to-peak amplitude of bursting (Fig 5D) and in the averaged integrated neurograms (Fig 5C). The effect of light on the locomotor-like activity was further characterized by examining the phasing between the activity of the bilateral flexor–dominated ventral roots (Fig 5E1) and that of the ipsilateral flexor–extensor ventral roots (Fig 5E2) in both *En1*-Arch and *En1*-GFP cords (Fig 5E; purple circles and black crosses, respectively). We found no statistical differences between the *En1*-Arch and the *En1*-GFP cords. However, in the *En1*-Arch cords, the ipsilateral flexor–extensor phasing during the light (large green circle; ϕ = 178.89, r = 0.9803) and after the light (large gray circle, ϕ = 159.12, r = 0.9057) was statistically different (Watson-Williams test, *p* = 0.0138).

Hyperpolarizing V1 interneurons also altered the pattern of the rhythm. Both the flexor-dominated and extensor-dominated burst durations increased under illumination, with the increase in the flexor burst durations being the most pronounced (Fig 5F1 and 5F2 left panel, green rectangles, flexor: 25.7% from 1.64 ± 0.4 s to 2.01 ± 0.45 s and extensor: 7.8% from 1.86 ± 0.28 s to 1.99 ± 0.4 s, Wilcoxon signed-rank test, *p* = 0.0002). The interburst interval also increased in the light, with the biggest changes in the extensor-dominated root (Fig 5F1 and 5F2, right panel, green rectangles, L5: 32% from 1.3 ± 0.34 s to 1.69 ± 0.46 s and L2: 11.9% from 1.44 ± 0.34 s to 1.59 ± 0.41 s, Wilcoxon signed-rank test, *p* = 0.0008), suggesting that a subset of V1 interneurons regulate both the flexor-dominated burst duration and the corresponding extensor-dominated interburst interval. The asymmetrical change in the burst and interburst interval durations during the light resulted in an alteration of the duty cycle for the ipsilateral flexor and extensor bursting activity (Fig 5F3). Under control conditions, the extensor-dominated root had a larger duty cycle than the flexor one (extensor: 0.59 ± 0.04, flexor: 0.53 ± 0.07, *p* < 0.0001), but in the light, their duty cycles converged and were not statistically different (extensor: 0.54 ± 0.04, flexor: 0.56 ± 0.06, *p* = 0.2075), and when the light was turned off, the duty cycles returned to the prelight control values (extensor: 0.58 ± 0.05, flexor: 0.53 ± 0.06, *p* < 0.0001).

Intracellular recordings from L5 motoneurons (Fig 6) revealed that the light-induced changes in the rhythmic synaptic drive were similar to those observed for dorsal root stimulation: an increase in the amplitude of the synaptic drive (Fig 6B and 6C1, *n* = 7, before illumination: 6.4 ± 3.52 mV; during illumination: 11.13 ± 4.5 mV; after illumination: 5.06 ± 3.01 mV; Friedman test, *p* = 0.0455), no change in the resting membrane potential (Fig 6C2, before illumination: −56.53 ± 9.12 mV; during illumination: −56.62 ± 9.3 mV; after illumination: −56.84 ± 9.15 mV; Friedman test, *p* > 0.9999), and an increase in the number of spikes per cycle (Fig 6C3, number of spikes: before illumination: 11.93 ± 10.19; during illumination: 22.58 ± 17.85; after illumination: 7.48 ± 7.18, Friedman test, *p* = 0.0455).

**Fig 7:**
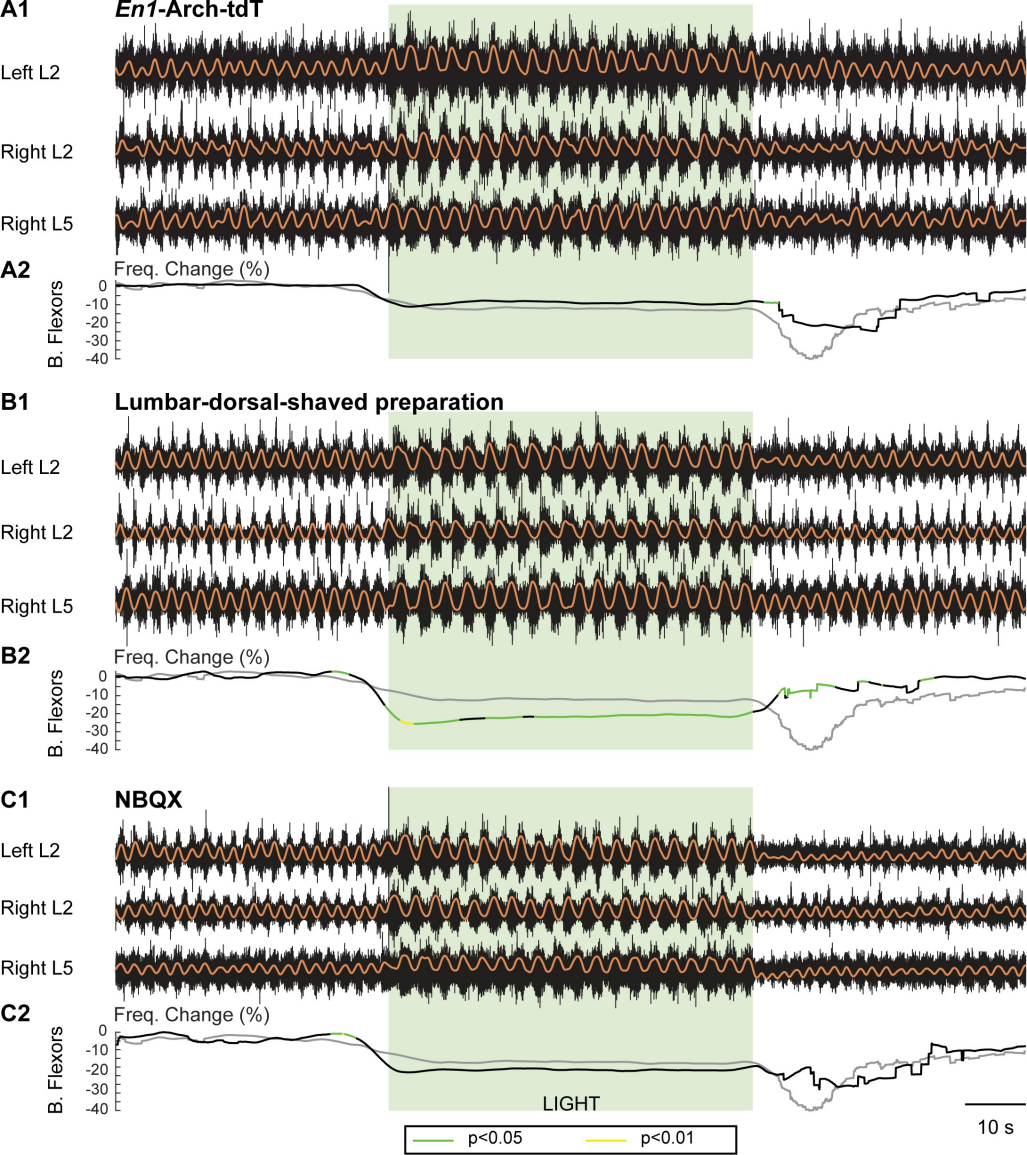
The light-induced decrease in frequency in *En1*-Arch cords is due to a ventral, nonglutamatergic mechanism and is similar in *En1*-Arch and *En1*-Arch-tdT spinal cords. (A1) Locomotor-like activity evoked by 5 μM NMDA, 10 μM 5-HT, and 50 μM DA in an *En1*-Arch-tdT spinal cord. The black traces show the high-pass (10 Hz) filtered signal from the left and right L2 and the right L5 ventral roots. The superimposed orange traces are the integrated neurograms. (A2) Time series showing the change in frequency averaged for all experiments for the bilateral flexor roots from *En1*-Arch-tdT preparations (black lines) and *En1-*Arch preparations (gray lines). The statistics were obtained using a bootstrap *t* test between *En1*-Arch-tdT (*n* = 6) and *En1*-Arch cords (*n* = 16). (B1) Locomotor-like activity evoked by 5 μM NMDA, 10 μM 5-HT, and 50 μM DA in an *En1*-Arch spinal cord lacking the lumbar dorsal horn. The black traces show the high-pass (10 Hz) filtered signal from the left and right L2 and the right L5 ventral roots. The superimposed orange traces are the integrated neurograms. (B2) Time series showing the change in frequency averaged for all experiments for the bilateral flexor roots from *En1*-Arch lumbar dorsal shaved preparations (black lines) and *En1-*Arch preparations (gray lines). The statistics were obtained using a bootstrap *t* test between *En1*-Arch dorsal shaved (*n* = 4) and *En1*-Arch cords (*n* = 16). (C1) Locomotor-like activity evoked by 7 μM NMDA, 10 μM 5-HT, 50 μM DA, in the presence of 5 μM NBQX in an *En1*-Arch spinal cord. The black traces show the high-pass (10 Hz) filtered signal from the left and right L2 and the right L5 ventral roots. The superimposed orange traces are the integrated neurograms. (B2) Time series showing the change in frequency averaged for all experiments for the bilateral flexor roots from *En1*-Arch with NBQX (black lines) and *En1-*Arch preparations (gray lines). The statistics were obtained using a bootstrap *t* test between *En1*-Arch dorsal shaved (*n* = 6) and *En1*-Arch cords (*n* = 16). *p*-Values are color coded as indicated in the box at the bottom of the figure. 5-HT, 5-hydroxytryptamine; Arch, archaerhodopsin-3; B. Flexor, bilateral flexor; DA, dopamine; *En1*, *engrailed-1*; Freq. Change, frequency change; NBQX, 2,3-dihydroxy-6-nitro-7-sulfamoyl-benzo[F]quinoxaline; NMDA, N-methyl-D-aspartate; tdT, tdTomato.

In preparations in which both tdT and Arch-GFP were expressed in V1 interneurons (see Fig 1), illumination led to a decrease in frequency (*n* = 6) that was similar to that in the *En1*-Arch preparations (Fig 7A). As V1 interneurons are ventrally located, we performed experiments on *En1*-Arch preparation while shaving the dorsal lumbar spinal cord (*n* = 5). We found again that the frequency of the locomotor-like rhythm decreased and the burst amplitude increased when hyperpolarizing V1 interneurons (Fig 7B). Furthermore, in *En1*-Arch preparations, while using 5 μM 2,3-dihydroxy-6-nitro-7-sulfamoyl-benzo[F]quinoxaline (NBQX) (antagonist of α-amino-3-hydroxy-5-methyl-4-isoxazolepropionic acid [AMPA] glutamatergic receptors; *n* = 6), we found that the decrease of the frequency was like spinal cords without the antagonist (Fig 7C), indicating that the hyperpolarized population is not releasing glutamate. Consistent with the intracellular recordings from *En1*-tdT-Arch neurons, these results indicate that V1 interneurons are hyperpolarized by light in the *En1*-Arch preparations.

### Function and activity of V1 interneurons in the hemisected spinal cord

**Fig 8:**
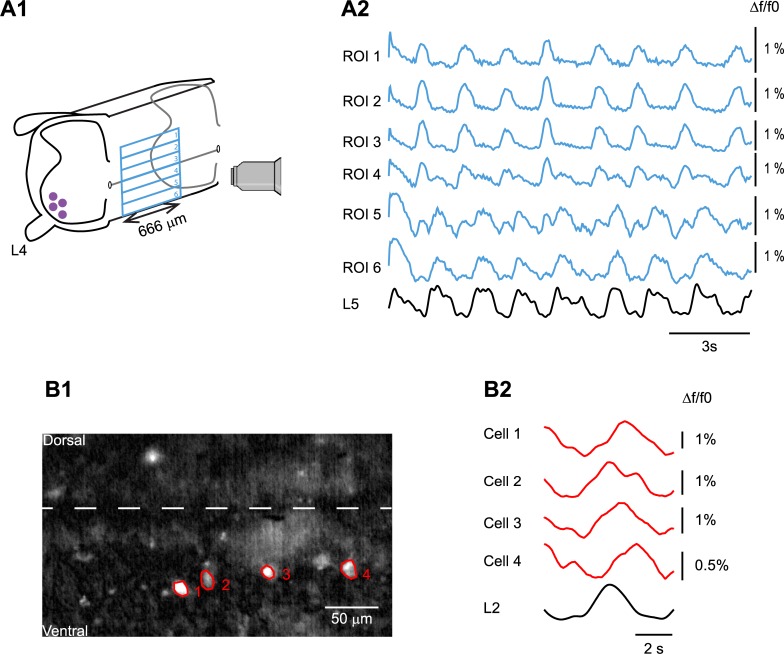
V1 interneurons are rhythmically active in hemisected spinal cords. (A1) Schematic of the experimental setup. Drawing of an *En1*-GCaMP6f hemisected spinal cord (in which V1 interneurons express the calcium indicator GCaMP6f) showing the lumbar L4 and rostral segments with the ventral side down. The filled purple circles represent motoneurons. The outer blue rectangle indicates the field of view of the 20× objective. The six smaller blue rectangles are ROIs. (A2) Calcium signals recorded from the six ROIs (blue traces) are shown together with the integrated neurogram of the L5 ventral root during rhythmic activity evoked by 5 μM NMDA, 10 μM 5-HT, and 50 μM DA in an *En1*-GCaMP6f hemisected spinal cord. (B1) Image of the cut face of a hemisected cord showing individual *En1*-GCaMP6f neurons, at a more restricted field of view from that shown in A1. The dotted line identifies the central canal and ROIs (red) that were placed around four neurons. (B2) Cycle-triggered calcium signals from the four cells identified in B1 (red traces) extracted after subtracting the local neuropil signal (see Methods) are shown together with the cycle-triggered, integrated neurogram of the L2 ventral root during rhythmic activity evoked by 5 μM NMDA, 10 μM 5-HT, and 50 μM dopamine in an *En1*-GCaMP6f hemisected spinal cord. The vertical scales on the right of the calcium signals represent the percentage change in fluorescence. 5-HT, 5-hydroxytryptamine; DA, dopamine, *En1*, *engrailed-1*; NMDA, N-methyl-D-aspartate; ROI, region of interest.

Previous work has shown that deletion of V2b neurons in a hemicord leads to synchronous activation of flexor and extensor motoneurons [12], implying that the V1 population cannot support flexor–extensor alternation under these conditions. One hypothesis to explain this observation is that the subset of V1 neurons responsible for flexor–extensor alternation is normally contralaterally activated [19]. Moreover, in the Zhang paper [12], the frequency changes accompanying lesioning of either V2b or V1 neurons in the hemicord were not examined. For these reasons, we were interested in determining whether the light-induced reduction in locomotor frequency we observed in the whole cord still occurred in the hemicord. Assuming some of the contralaterally driven V1 neurons are inactive, then the effects of light would be due to its action on those neurons that remain active in the hemicord. Renshaw cells have been shown to be driven primarily by motoneurons [20,21] and hence should be active in a hemicord. Ia inhibitory interneurons have also been shown to be rhythmically active during locomotor-like activity [21,22] and have been hypothesized to be driven by rhythmic excitatory inputs [21] and therefore should also be active in a hemicord. To determine whether V1 neurons are active in the hemicord—other than Renshaw cells or Ia inhibitory interneurons—we used *En1*-GCaMP6f preparations, which express selectively GCaMP6f (a genetically encoded calcium indicator) in V1 interneurons, to visualize their activity during drug-induced fictive locomotion. To do this, we imaged the cut, medial face of the cord to minimize the contribution of Renshaw cell or Ia inhibitory interneuron activity to the optical signals. Rhythmic activity was elicited from the hemicords using 5 μM NMDA, 10 μM 5-HT, and 50 μM DA. To quantify the optical signals, the medial surface of the hemicords was subdivided dorsoventrally into six regions of interest (ROIs) of the same size (Fig 8A1). In all experiments (seven out of seven), rhythmic calcium activity at the same frequency as the extracellular ventral root recordings was observed. In five out of seven experiments, the imaging revealed rhythmic calcium transients both in phase (Fig 8A2, ROI 6) and out of phase (Fig 8A2, ROI 1–3) with the ipsilateral ventral root electrical signal. In some regions (Fig 8A2, ROI 4,5), the optical signals contained both in- and out-of-phase components. Furthermore, in four out of seven experiments, we could resolve individual, rhythmically active neurons located close to the medial surface of the hemicord (Fig 8B). Because these neurons are located close to the most medial aspect of the cord, they are unlikely to be either Renshaw or Ia inhibitory interneurons [2,23–26]. Collectively, these experiments suggest that several classes of V1 interneuron are active in hemisected spinal cords in addition to Renshaw cells and Ia inhibitory interneurons.

**Fig 9:**
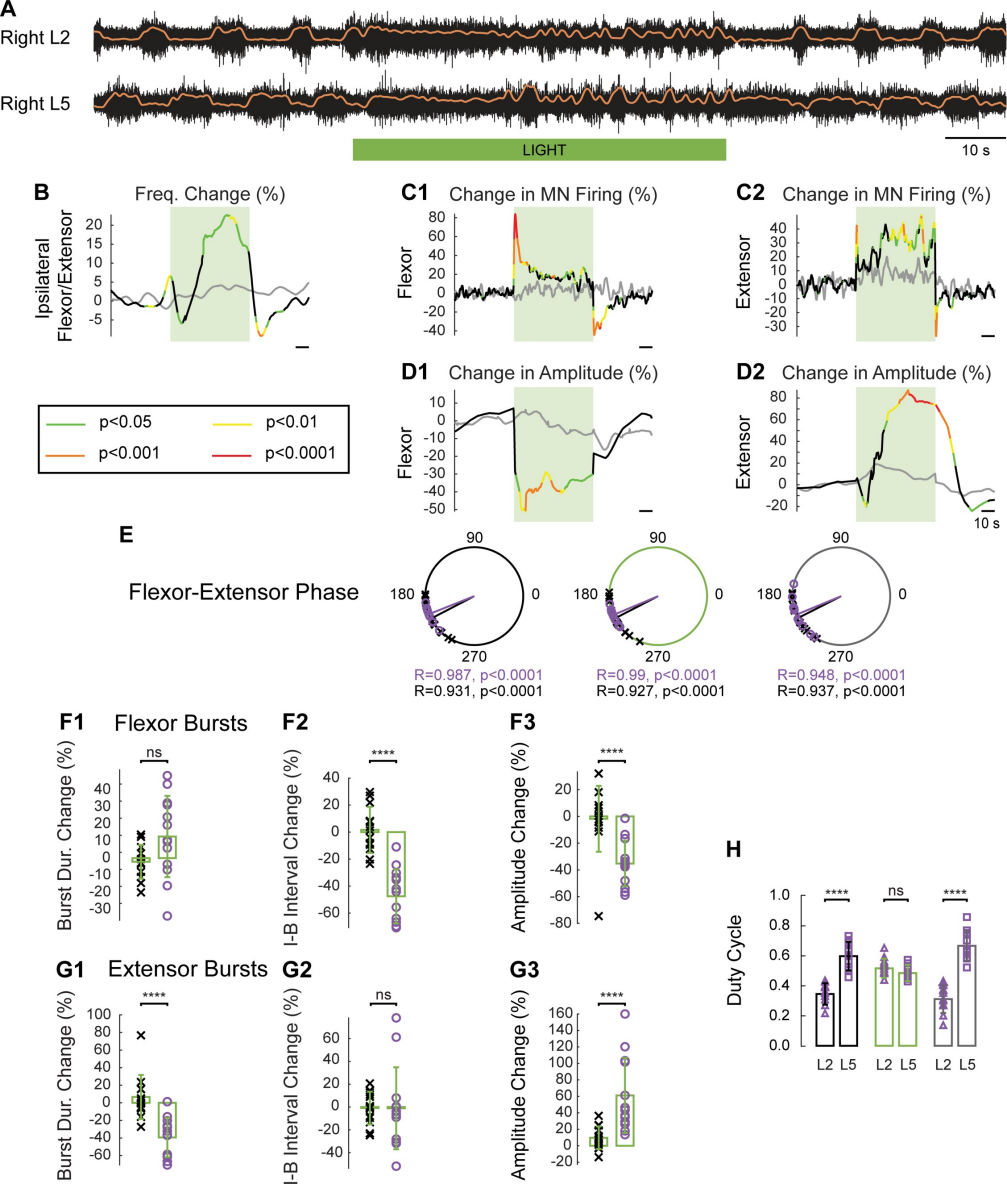
During drug-induced motor activity in the hemisected spinal cord, light-induced hyperpolarization of V1 interneurons accelerates the rhythm and changes its pattern. (A) Rhythmic activity evoked by 5 μM NMDA, 10 μM 5-HT, and 50 μM DA in an *En1*-Arch hemisected spinal cord. The black traces show the high-pass (10 Hz) filtered signal recorded from the right L2 and L5 ventral roots. The superimposed orange traces are the integrated neurograms. The green bar indicates the duration of the light (60 s). (B) Time series showing the change in frequency averaged for all experiments for the ipsilateral flexor/extensor roots of the *En1*-Arch hemisected cords (black trace) versus WT hemisected cords (gray traces). (C) Change (%) in the averaged integrated ventral root discharge (Change in MN Firing) for the ipsilateral flexor (C1) and extensor (C2) ventral roots of the *En1*-Arch hemisected cords (black trace) versus the WT hemisected cords (gray traces). (D) Time series showing the change (%) in the averaged amplitude (trough to peak) of the integrated ventral root discharge (Change in Amplitude) for the ipsilateral flexor (D1) and extensor (D2) ventral roots for the hemisected *En1*-Arch preparations (black lines) versus hemisected *En1-*GFP preparations (gray lines). (B–D) The statistics were obtained using a bootstrap *t* test between *En1*-Arch (*n* = 13) and *En1*-GFP cords (*n* = 13), and the *p*-values are color coded as indicated in the box in the lower-left panel. (E) Circular plots showing the phasing of the ipsilateral flexor–extensor ventral roots during fictive locomotion before (large black circle), during (large green circle), and after (large gray circle) illumination in WT (black crosses) and *En1*-Arch (small purple circles) hemicords. R is the length of the vector, and *p* is the value for the Rayleigh test of uniformity. Using the Harrison-Kanji test, we calculated the statistical differences between the two groups of animals (genetic Identity) and the differences between light on and light off (light status) for the phasing of the ipsilateral L2 and L5 root activity. The results of the tests were light status: F(2, 72) *p* = 0.9686, genetic identity: F(1, 72) *p* = 0.1966, and interaction: F(2, 72) *p* = 0.9413. (F) Light-dependent changes in the characteristics of the flexor-related ventral root bursts. Bar plot showing the change (%) in the Burst Dur. (F1), the I-B interval (F2), and the burst amplitude (F3) during illumination (green rectangles) compared with before the light in WT (black crosses) and *En1*-Arch (purple circles) hemicords. (G) Same plots as F, for the extensor-related ventral root bursts. Two-way ANOVA was used to statistically compare the Burst Dur. between L2 and L5 in WT and *En1*-Arch (light status: F[2, 144] *p* < 0.0001, genetic identity: F[3, 144] *p* = 0.4215, interaction: F[6, 144] *p* < 0.0001). The same test was used to compare the I-B interval duration (light status: F[2, 144] *p* < 0.0001, genetic identity: F[3, 144] *p* = 0.2704, interaction: F[6, 144] *p* < 0.0001) and the amplitude change (light status: F[2, 144] *p* = 0.0267, genetic identity F[3, 144] *p* < 0.0001, interaction: F[6, 144] *p* < 0.0001). (H) Duty cycle of the L2 (purple squares) and L5 (purple triangles) cycles in *En1*-Arch hemisected cords before (black rectangles), during (green rectangles), and after (gray rectangles) illumination (two-way RM ANOVA, light status: F[2, 24] *p* = 0.0187, roots: F[1, 12] *p* < 0.0001, interaction: F[2, 24] *p* < 0.0001). *p < 0.05, **p < 0.01, ***p < 0.001, ****p < 0.0001. The data underlying this figure can be found in S6 Data. 5-HT, 5-hydroxytryptamine; Arch, archaerhodopsin-3; Burst Dur., burst duration; DA, dopamine, *En1*, *engrailed-1*; Freq. Change, frequency change; GFP, green fluorescent protein; I-B, interburst; MN, motoneuron; NMDA, N-methyl-D-aspartate; ns, not significant; RM, repeated measures; WT, wild-type.

Given that some classes of V1 interneuron were active in the hemicords, we then examined the effect of green light on the rhythmic activity generated in the *En1*-Arch hemicords. For these experiments, we used wild-type (WT) cords as the controls because the effects of light on the rhythmic activity were similar in WT and *En1*-GFP cords (S4 Fig). Rhythmic activity evoked with 5 μM NMDA, 10 μM 5-HT, and 50 μM DA (Fig 9A and S5 Fig) was slower than that observed in the full cord (*En1*-Arch: whole-cord frequency: 0.33 ± 0.06 Hz, hemicord frequency: 0.11 ± 0.02 Hz, Mann-Whitney test *p* < 0.0001, WT: whole-cord frequency: 0.36 ± 0.06 Hz, hemicord frequency: 0.13 ± 0.03 Hz, Mann-Whitney test *p* < 0.0001), as previously reported [17]. However, we were surprised to find that hyperpolarizing V1 interneurons resulted in an initial period of tonic activity, which was followed by rhythmic activity that was faster than that recorded before the light (Fig 9B); when the frequency was calculated from just the rhythmically active part of the signal, it increased from 0.10 ± 0.02 to 0.17 ± 0.06 Hz, Friedman test, *p* < 0.0001 (by 2.1% to 175.6% for an average increase of 75.7% ± 59.1%, *n* = 13), whereas in the WT hemicords, the frequency did not change (from 0.14 ± 0.03 to 0.14 ± 0.03, *n* = 13, Friedman test, *p* = 0.7939, Fig 9B gray line and S5 Fig). After the light was turned off, there was a transient decrease in the frequency of the rhythm that was statistically different from the postlight frequency in WT hemicords (Fig 9B, *p* < 0.01). During the light, the firing of motoneurons was increased in both the flexor-dominated and extensor-dominated roots, particularly at the onset of the light in the flexor-dominated roots (Fig 9C). However, when we examined the burst amplitude (trough to peak) of the integrated neurogram, we found that the flexor-dominated ventral root showed a decrease in amplitude (−35.25% ± 17.11%), whereas the extensor-dominated ventral root showed an increase in amplitude (61.14% ± 45.54%), and this was statistically different from the WT hemicords (two-way ANOVA, *p* < 0.0001). In the hemicords, the phasing of the activity stayed stable in both WT and *En1*-Arch cords during and after the light (Fig 9E).

During the light, the pattern of activity in the flexor-dominated and extensor-dominated ventral roots was altered. When V1 interneurons were hyperpolarized, there was a decrease in the extensor burst duration (−39.39% ± 22.36% from 6.28 ± 1.77 s to 3.62 ± 1.23 s; Fig 9G1, green rectangle) with little change in the flexor burst duration (12.68% ± 23.85% from 3.78 ± 1.01 s to 4.08 ± 1.26 s; Fig 9F1, green rectangle). This was accompanied by a statistically significant decrease in the flexor interburst interval (−47.57% ± 20.08% from 6.6 ± 1.81 s to 3.3 ± 1.08 s; Fig 9F2, green rectangles), suggesting that in the hemicord, V1 neurons regulate the extensor-dominated burst duration and the corresponding flexor-dominated interburst interval. This is different from the behavior in the whole cord, in which the V1 neurons influence primarily the flexor-dominated burst duration and the extensor-dominated interburst interval. These light-induced changes resulted in a duty cycle that was similar for the two roots (flexor: 0.52 ± 0.06, extensor: 0.49 ± 0.05, two-way repeated measures [RM] ANOVA, *p* = 0.239) and which differed from the values both before (flexor: 0.35 ± 0.06, extensor: 0.61 ± 0.09, *p* < 0.0001 for both) and after the light (flexor: 0.32 ± 0.09, extensor: 0.68 ± 0.09, *p* < 0.0001 for both) when the duty cycle in the extensor-dominated ventral root was significantly longer than that in the flexor-dominated ventral root (Fig 9H). These findings, together with the observation that the flexor-dominated burst amplitude decreased during the light, whereas the extensor-dominated burst amplitude increased (Fig 9D, 9F3 and 9G3, green rectangles), suggest that V1 interneuron behavior differs in the hemicord and the whole cord (S6 Fig), which could occur at the level of motoneurons or premotor interneurons or within the locomotor CPG itself.

**Fig 10:**
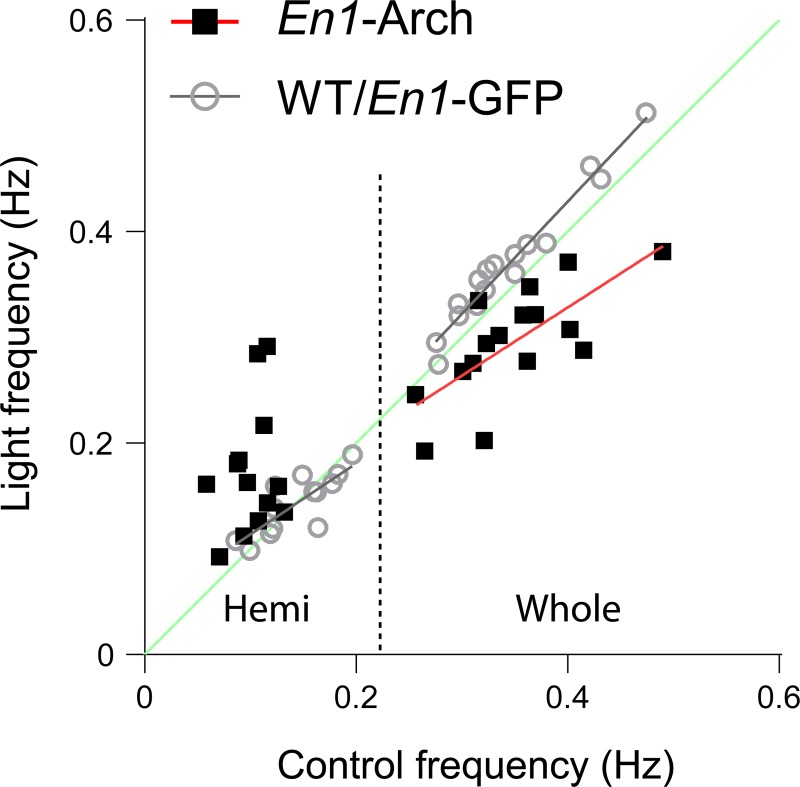
Relationship between the initial frequency and the effect of the light in *En1*-Arch and WT/*En1*-GFP preparations during rhythmic activity evoked by NMDA, 5-HT, and DA. Plot showing the frequency of the rhythm evoked by NMDA, 5-HT, and DA before the light (Control frequency) plotted against the frequency of the rhythm during the light (Light frequency) in *En1*-Arch (black squares) and WT/*En1*-GFP (gray circles) cords. The green line represents the line of unity, in which the frequency is the same before and during light. The red line is the linear regression calculated for the *En1*-Arch experiments in whole cords, whereas the gray lines are the linear regressions calculated separately for the whole cords and hemicords from WT/En1-GFP animals. The hemisected data and the whole-cord data are separated by the dotted line. WT/*En1*-GFP (hemicord *n* = 13; Spearman correlation r = 0.9009 *p* = 0.001, slope: 0.6694x + 0.04695; whole cord *n* = 16; Spearman correlation r = 0.9788 *p* < 0.0001, slope: 1.062x + 0.003816); *En1*-Arch (hemicord *n* = 13; Spearman correlation r = 0.2469 *p* = 0.4162; whole cord *n* = 16; Spearman correlation r = 0.7213 *p* = 0.016, slope: 0.6439x + 0.07074). Difference between the slopes: WT/*En1*-GFP whole-cord slope versus *En1*-Arch whole-cord slope (F[1, 28] = 5.470, *p* = 0.0267). The data underlying this figure can be found in S7 Data. 5-HT, 5-hydroxytryptamine; Arch, archaerhodopsin-3; DA, dopamine; *En1*, *engrailed-1*; GFP, green fluorescent protein; Hemi, hemicords; NMDA, N-methyl-D-aspartate; WT, wild-type.

Because the frequency of the rhythm was much slower in the hemicords, it is possible that the direction and magnitude of the light-induced changes in frequency depended on the initial prelight, control frequency of the rhythm. To address this idea, we plotted the initial control frequency (prelight) against the frequency during the light for WT/*En1*-GFP and *En1*-Arch preparations (Fig 10). In whole WT/*En1*-GFP cords, light accelerated the rhythm slightly (Fig 10, gray line), whereas in hemicords, it was centered around the unity line (Fig 10, green line). In *En1*-Arch whole-cord preparations, the regression line (red line) was tilted to the right of the line of identity (green line) such that the biggest decreases in frequency occurred at the fastest rhythms, indicating that the light-induced changes in frequency are proportional to the control frequency. The frequencies for the *En1*-Arch hemicords did not show a significant correlation; therefore, no regression line was plotted. However, all hemicords showed a slow rhythm before the light, and the frequency of the rhythm accelerated upon hyperpolarization of V1 interneurons.

## Discussion

We have shown that manipulating firing of the V1 neuronal population has potent effects on the locomotor rhythm generator in both intact and hemisected cords. We confirmed earlier work [6] showing that deleting or acutely hyperpolarizing V1 neurons slowed the locomotor rhythm during drug-induced locomotion and extended the previous observations by showing that light-induced slowing also occurred during locomotor-like activity evoked by dorsal or ventral root stimulation. We found, however, that hyperpolarization of this population produced several other effects on the rhythm that have not been documented previously. These included an increase in the amplitude of the locomotor drive potentials, a change in the pattern of ventral root activity, and the demonstration that some V1 interneurons are active in hemicords. In addition, we have shown that changes in the frequency and pattern induced by hyperpolarizing the V1 population are more complex than previously reported, suggesting the existence of specialized subsets of V1 interneurons with distinct functions.

### Do V1 interneurons mediate the effects of ventral root stimulation on the locomotor CPG?

Previously, we showed that ventral root stimulation can activate the locomotor CPG [13,27] and that the frequency of the rhythm slowed when motoneurons were optogenetically hyperpolarized [18]. These effects could be mediated through motoneuronal projections to Renshaw cells because hyperpolarizing V1 interneurons, including Renshaw cells, also reduced the frequency of the drug-induced locomotor-like rhythm. However, V1 hyperpolarization did not block activation of the locomotor rhythm by ventral root stimulation, suggesting that the effect is not mediated by motoneuronal projections to Renshaw cells. One caveat to this conclusion is that we do not know the degree to which Renshaw cells—or, for that matter, any of the V1 subpopulations—are hyperpolarized during the light. Intracellular recordings from 13 identified V1 neurons showed an average hyperpolarization of approximately 9 mV. However, somatic recordings provide no information about the level of hyperpolarization in V1 axons or their terminals. Nevertheless, it is clear we are hyperpolarizing a significant fraction of the V1 population because we were able to reproduce the effects of ablating V1 interneurons on the frequency of the rhythm.

### V1 influences on the frequency of the locomotor rhythm

*En1*-positive neurons in the tadpole and the zebrafish have been shown to be involved in fast swimming [7,9]. Recently, in the zebrafish, it was shown that two classes of V1 interneurons (slow and fast) are selectively activated during slow and fast swimming, respectively [8]. Furthermore, it was hypothesized that V1 interneurons may also be involved during fast locomotion in the mouse because chemical hyperpolarization or genetic inactivation of V1 interneurons slows the locomotor rhythm [6,12]. In the present work, we have shown that hyperpolarization of V1 interneurons affects the frequency of the rhythm in a more complex fashion than previously described. In the *En1*-Arch whole-cord preparations, the rightward tilt of the regression line between the prelight frequency and the frequency in the light indicates that the light-induced changes in frequency are proportional to the prelight frequency, with the largest decreases associated with the highest frequencies. One way to account for this is to propose that the V1 neurons responsible for these effects are progressively recruited as locomotor speed increases. Therefore, proportionately more of these V1 neurons would be available to hyperpolarize as the frequency increases.

The maximum light-induced frequency changes we observed were smaller than those previously reported [6]. There are several reasons why this could have occurred. In the earlier studies, in vitro locomotor-like activity was produced using NMDA and 5-HT, whereas in our work we used NMDA, 5-HT, and DA, which produces a slower rhythm. Furthermore, the presence of DA may recruit different circuit components that might moderate the frequency changes. The remaining reasons are technical. Light induced an increase in the frequency in WT cords, possibly because of tissue heating (see also [18]). Because this effect is likely to be present in the *En1*-Arch cords, it would have mitigated the light-induced frequency decrease. It is also possible that the V1 population was incompletely silenced by the light.

In contrast to the effects of light on the whole cord, light increased the frequency of the rhythm after a period of tonic activity in the hemicords. This could be explained by the existence of two subtypes of V1 interneuron that are responsible for the acceleration and deceleration of the rhythm when hyperpolarized—the former being ipsilaterally driven, whereas the latter is driven contralaterally. In whole cords, the contralaterally driven population must therefore inhibit the ipsilaterally driven (accelerating) population to allow for the slowing of the frequency. This architecture was proposed by Shevtsova and Rybak [19], who argued that two classes of V1 neurons operate at the level of the rhythm generator. One class, V1-1, receives excitatory input from the extensor half-center and inhibits the flexor half-center. The other V1 class is normally driven by tonic contralateral input, inhibits the extensor half-center, and projects disynaptic inhibition to the flexor half-center through a non-V1 inhibitory neuron. In the hemicord, the tonically driven V1 neuron population is inactive, which disinhibits the non-V1 inhibitory neuron population projecting to the flexor half-center, thereby slowing the rhythm. When the light is turned on, the V1-1 subgroup is hyperpolarized, which removes inhibition from the flexor center, thereby accelerating the rhythm.

It is possible that this mechanism also explains the initial period of tonic activity that precedes the accelerated rhythm. If the flexor rhythm generator is composed of pacemaker-like cells, then the removal of V1-1 inhibition might depolarize the cells into a voltage regime in which they become tonically active [28]. As the removal of inhibition recovers—due perhaps to a decay of the optogenetically induced hyperpolarization (see Fig 2B2)—the depolarization is reduced, and the cells return to the bursting regime. Whether the light-induced hyperpolarization of the hypothesized V1-1 neurons is enough to depolarize the flexor rhythm generator into tonic firing must be established or refuted by further experiments.

Our finding of a light-induced acceleration of the rhythm in hemicords contrasts with the findings of Zhang and colleagues [12], who showed that the frequency of rhythmic activity was similar in WT hemicords and hemicords in which V1 neurons had been genetically silenced. The difference between our results and those of Zhang and colleagues [12] might be because they performed the hemisection in cords in which V1 function was absent. It is possible, therefore, that some network compensation or reorganization may have been present in these cords that would not have occurred when V1 neurons are acutely hyperpolarized.

### Regulation of the duration of flexor and extensor bursts by V1 interneurons

V1 hyperpolarization of the whole cord lengthened the flexor-related bursts and the extensor-related interburst interval by approximately 40%. This is consistent with earlier work indicating that V1 interneurons preferentially inhibit flexor motoneurons during locomotion [4]. The coupled effects of V1 hyperpolarization on flexor burst duration and extensor interburst interval are most simply explained by the actions of V1-derived, Ia inhibitory interneurons. During fictive locomotion, the Ia inhibitory interneurons that are rhythmically coactive with either flexor or extensor motoneurons project to their antagonist motoneurons and contribute inhibition to the inactive phase of their cycle [29]. In this way, the duration of activity of the motoneurons and their corresponding Ia inhibitory interneurons will be correlated with the interburst interval of the antagonist motoneurons.

However, we also observed smaller increases in the duration of the extensor-related bursts and the coupled flexor-related interburst interval. Because V2b-derived, Ia inhibitory interneurons regulate the duration of the extensor bursts during locomotion [4], it is unclear what accounts for the increased duration of extensor bursts when V1 neurons are hyperpolarized. Although V1 neurons mainly innervate flexor motoneurons, they also innervate extensor motoneurons, albeit to a lesser extent [4]. Therefore, the simplest assumption to account for our data is that V1-derived Ia inhibitory interneurons regulate the extensor burst duration to a lesser extent than the flexors because of the relative strength of their projections to flexor and extensor motoneurons.

Furthermore, in the hemicord, V1 hyperpolarization produced a much smaller increase in the flexor burst duration (approximately 10%) than occurred in the whole cord. More striking, however, were the effects on the extensor burst durations and the flexor interburst intervals, which both decreased by approximately 40%–50%. We do not know why this behavior differs from that in the whole cord, but it presumably results from the loss of contralateral inputs. If the V1- and V2b-derived Ia inhibitory interneurons neurons are mutually inhibitory, as has been shown for flexor- and extensor-related Ia inhibitory neurons in the adult cat [30], then V1 hyperpolarization will result in less inhibition of the V2b-Ia interneurons, thereby enhancing the V2b inhibition of extensor motoneurons. This could also occur in the whole cord and thereby explain the slight increase in the extensor-dominated burst duration. The differential effects of V1 inhibition on the duration of flexor and extensor bursts might also be influenced by non-Ia, V1 projections to the CPG as formulated in models [19], or to other premotor interneurons [12].

Studies on nonresetting deletions [31,32] have shown that the pattern of locomotor-like activity can be altered while the frequency remains unchanged, leading to a model of a CPG with two layers: a rhythm-generating layer and an independent pattern-generating layer [33]. It is known that the durations of the flexor and extensor bursts are correlated with the frequency of the rhythm in vivo [34] and in vitro with a limb-attached preparation [35,36] such that increases in frequency are accompanied by a shortening of the burst durations, particularly in extensor motoneurons. In the isolated rat spinal cord, however, this asymmetry is only observed when the limb afferents are intact and the limb can move [35]. In the deafferented, isolated cord, both flexors and extensors show a similar decrease in the burst duration with increasing frequency [35]. The relative frequency independence of the extensor burst durations involves an interaction between afferents and V1 interneurons because it is abolished during air stepping, in mice in which V1 neurons are functionally silenced [4].

We observed a small but significant difference between the flexor and extensor duty cycles in the whole cord. However, in hemicords, there was a clear asymmetry between them. Therefore, an effect on the pattern would be better assessed in the hemicord. Consistent with this idea, when V1 interneurons were hyperpolarized, the differences in the flexor-related and extensor-related duty cycles were almost abolished. This suggests that, even in the absence of limb-driven afferent inputs, V1 interneurons are still mediating the asymmetrical activation of flexor and extensor motoneurons. These observations raise the possibility that, in the absence of V1 function, the default state of the CPG is symmetrical, with antagonist motoneurons each firing with an approximately 50% duty cycle. We hypothesize that one function of the V1 population is to set the burst durations of muscles to be appropriate to their biomechanical function and to adapt to environmental demands such as changes in locomotor speed. This idea could be tested in future experiments by examining the role of V1 interneurons in determining the specialized bursting patterns of individual motoneuron pools during locomotor-like activity [37].

## Methods

### Animals

All experiments were carried out in compliance with the National Institute of Neurological Disorders and Stroke Animal Care and Use Committee (Animal Protocol Number 1267).

Experiments were performed on Swiss Webster WT (Taconic Laboratory) or transgenic mice between the day of birth to postnatal day 5 (P0–P5). Only two preparations were obtained from P8 and P10 animals (for intracellular recordings of putative V1 interneurons). We used several different transgenic mouse lines in the experiments: Arch-GFP (Arch, B6;129S-*Gt[ROSA]26Sor*^*tm35*.*1[CAG-aop3/GPF]Hze*^/J, stock# 012735), floxed EGFP (EGFP, *Gt[ROSA]26Sor*^*tm1*.*1[CAG-EGFP]Fsh*^/Mmjax, MMRRC stock# 32037-JAX), floxed tdT (tdT, B6.Cg-*Gt[ROSA]26Sor*^*tm9[CAG-tdTomato]Hze*^/J, stock# 007909), and GCaMP6f (GCaMP6f, B6;129S-*Gt[ROSA]26Sor*^*tm95*.*1[CAG-GCaMP6f]Hze*^/J, stock# 024105) were obtained from Jackson Laboratories, whereas *En1-*cre was kindly gifted by Martyn Goulding.

### Dissection

The mice were decapitated and eviscerated and then placed in a dissecting chamber and continuously superfused with aCSF (concentrations in mM: 128.35 NaCl, 4 KCl, 1.5 CaCl_2_.H_2_O, 1 MgSO_4_.7H_2_O, 0.58 NaH_2_PO_4_.H_2_O, 21 NaHCO_3_, 30 D-glucose) and bubbled with 95% O_2_ and 5% CO_2_. For the two older preparations, mice were first anaesthetized with isoflurane; once they were nonresponsive to tail pinching, they were decapitated, eviscerated, and placed in the dissection chamber and continuously perfused with a cold oxygenated low-calcium, high-magnesium aCSF (concentrations in mM: 128.35 NaCl, 4 KCl, 0.5 CaCl_2_.H_2_O, 6 MgSO_4_.7H_2_O, 0.58 NaH_2_PO_4_.H_2_O, 21 NaHCO_3_, 30 D-glucose). After a ventral laminectomy, the cords were isolated together with the attached roots and ganglia and left at room temperature. For experiments with intracellular recordings, the pia was removed either on the ventral part of the left L5 segment or on the lateral side of the right L5 segment.

### Electrophysiological recordings

Motoneuron extracellular activity was recorded with plastic suction electrodes into which individual ventral roots were drawn. Recordings were obtained from three lumbar ventral roots, two from the flexor-dominated ventral roots (either L1 or L2: left and right), and one from an extensor-dominated ventral root (L5: left or right). Locomotor-like activity was elicited by stimulating a sacral dorsal root ganglion, a sacral dorsal root, or a ventral root (4 Hz, train duration: 10 s, stimulus duration: 250 μs). The lowest intensity at which the train elicited locomotor-like activity was defined as the threshold (T), and stimulation was performed at 2 × T. Locomotor-like activity was also elicited by applying a drug cocktail: 5 μM NMDA, 10 μM 5-HT, and 50 μM DA. In one set of experiments, we added NBQX disodium salt (5 μM) to a 7 μM NMDA, 10 μM 5-HT, and 50 μM DA drug cocktail.

In preparations in which we removed the lumbar dorsal horn (dorsally shaved, Fig 7), the lumbar dorsal pia was first removed. The spinal cord was then pinned dorsal up onto a strip of beeswax mounted on a small piece of plexiglass and transferred to the vibratome chamber containing an ice slush, oxygenated K-gluconate–based solution (concentrations in mM: 130 K-gluconate, 15 KCl, 0.05 EGTA, 20 HEPES, 25 D-glucose, 1 mM kynurenic acid, 2 mM Na-pyruvate [adjusted to pH 7.4 with KOH]). The dorsal part of the lumbar spinal cord (approximately 250 μM, or until the central canal was visible) was then removed with a razor blade mounted on the vibratome. The cord was then transferred to a recording chamber with aCSF at room temperature and allowed to recover for at least 30 min. Subsequent recordings were made in the same manner as for the whole cord. We also performed experiments in which we hemisected the spinal cord. For this purpose, a thin pin was used to transect the spinal cord, and only one-half of the cord was used in the experiment. In these experiments, only the ipsilateral L2 and L5 ventral roots, reflecting flexor and extensor activity, respectively [17], were recorded. Motor activity was evoked by applying NMDA, 5-HT, and DA. In all experiments, the signals were amplified (1,000 times), band-pass filtered between 0.1 Hz and 3 kHz, and digitized at 10 kHz (Digidata 1322A, 1440A, 1500; Molecular Devices, Sunnyvale, CA, United States) and stored for further analysis. NMDA, 5-HT, DA, and kynurenic acid were obtained from Sigma-Aldrich (St. Louis, MO, USA), whereas NBQX was obtained from Tocris (Minneapolis, MN, USA).

### Intracellular recordings

Whole-cell recordings were obtained from motoneurons or interneurons with patch electrodes pulled from borosilicate capillaries with a microelectrode puller (model P-80 or P-97, Sutter instruments, Novato, CA, USA). Two different preparations were used to perform whole-cell patch-clamp recordings. After removing the pia matter, pipettes (resistance: 3–9 MΩ) were advanced blindly into the ventral horn of the spinal cord. Patch electrodes were filled with intracellular solution containing 10 mM NaCl, 130 mM K-gluconate, 10 mM HEPES, 11 mM EGTA, 1 mM MgCl_2_, 0.1 mM CaCl_2_, and 1 mM Na_2_ATP (pH adjusted to 7.2–7.4 with KOH). In another set of experiments, using the *En1*-tdT-Arch animals, the dorsal part of the lumbar spinal cord was removed to gain access to ventrally located interneurons. As described earlier, the lumbar dorsal pia was removed, and the cord then was transferred to the vibratome chamber containing an ice slush, oxygenated low-calcium, high-magnesium aCSF. Interneurons were then visually targeted with glass pipettes (resistance: 3–5 MΩ) filled with an intracellular solution containing 10 mM NaCl, 130 mM K-gluconate, 10 mM HEPES, 1.1 mM EGTA, 1 mM MgCl_2_, 0.1 mM CaCl_2_, and 1 mM Na_2_ATP and 0.5% Neurobiotin (pH adjusted to 7.2–7.4 with KOH). Neurons were only considered for subsequent analysis if they exhibited a stable resting membrane potential and, for motoneurons, if they exhibited an overshooting antidromically evoked action potential. The input resistance was calculated from the slope of the current/voltage (200 ms) plot within the linear range. The liquid junction potential was not corrected. All recordings were obtained with a Multiclamp 700A or 700B (DC-3kHz, Molecular Devices).

### Activation of the opsins

To activate Arch, we illuminated the cord ventrally with a continuous green light (540–600 nm) from a 3-mm-diameter light guide connected to a light-emitting diode (LED, X-cite, Excelitas, Waltham, MA, USA). As previously described [18], all the lumbar segments were illuminated. We illuminated the cords with the maximum light intensity because we showed in a previous study that the stronger intensity provides the more-stable results [18]. The same protocol was applied to mice devoid of the opsins (WT or *En1*-GFP) in control experiments to establish whether the light itself had any effects on the evoked locomotor-like activity. For all animals, when evoking locomotor-like activity with stimulation, 3–5 trials with and without the light were obtained. For drug-induced fictive locomotion, 3-min trials were used (3–7 trials) comprising 60 s prelight, 60 s with the light on, and 60 s postlight.

### Electrophysiology analysis

To compare recordings across trials and experiments, they were aligned to the beginning of the light during drug-induced locomotor-like activity, keeping 55 s before and after the light for the analysis. For stimulus-evoked episodes, the recordings were aligned to the first stimulus, keeping 1 s before the first stimulus and 4.5 s after the last stimulus for a total of 15.5 s. The signals were then analyzed using wavelet analysis [18,38]. All extracellular recordings were integrated by first low-pass filtering the records at 200 Hz, followed by high-pass filtering at 10 Hz, rectification, and then low-pass filtering at 5 Hz for drug-induced fictive locomotion or at 20 Hz for stimulus-evoked fictive episodes.

Wavelet spectrograms (3,200 points along the time dimension) were then generated from 3–7 trials. As the wavelet analysis produces artifacts (edge effects), 10 s at the beginning and the end of the spectrograms was removed. Times series of frequency as well as of the phasing between the bilateral flexor–dominated ventral roots (L1/2) and between the ipsilateral flexor–dominated (L1/2) and extensor–dominated L5 ventral roots were extracted from the spectrograms. We then calculated the mean values of frequency and phasing before, during, and after the light (for drug-induced locomotor-like activity only). The frequency time series was then normalized by dividing by the average frequency before the light was turned on, and 1 was subtracted to set the control value to an average of 0. It was subsequently multiplied by 100 to be expressed as a percentage change. This allowed us to monitor the changes occurring upon illumination and making the time series independently of the initial frequency, allowing us to compare the effect of the light across trials, experiments, and animals. A negative value reflects the percentage decrease in the frequency, whereas a positive value reflects the percentage increase in the frequency. For drug-induced fictive locomotion, the integrated neurograms were resampled at 19 Hz to keep the same timescale as the wavelet analysis. The integrated neurograms were also normalized to 0 during the control period. We then averaged the normalized time series for all the trials per experiment, and this average was then averaged across all experiments.

To compare statistically the time series, we used a bootstrap *t* test [18]. The bootstrap was iterated 10,000 times. We then plotted the two conditions that were compared and color coded the results with black for not statistically significant, green for *p* < 0.05, yellow for *p* < 0.01, orange for *p* < 0.001, and red for *p* < 0.0001.

We also determined the troughs and the peaks of the integrated neurograms. We defined the beginning and the end of a burst as 40% of the distance between the trough and the peak. Once calculated, we could then extract the duration of the bursts and the interburst interval durations. We also calculated the amplitude of the burst (peak–trough). To compare the values between trials and experiments, we averaged the variables per trial before the light, during the light, and after the light (for the drug-induced fictive locomotion only). The variables were then normalized by setting the average before the light to 0. We also averaged all the cycle measurements across trials to get one value per experiment and then averaged the resulting values across experiments. All statistics were done using Prism 8.0 (GraphPad software, La Jolla, CA, USA), except for the circular statistics, which was done with the Matlab (Mathworks, Natick, MA, USA) circular statistic package (https://github.com/circstat/circstat-matlab, [39]). To compare the mean values under the different conditions (light on/off–light status; animal type–genetic identity), we used nonparametric statistics (Friedman, Wilcoxon, Mann-Whitney, and Kruskal-Wallis tests) or two-way ANOVA with a two-stage linear step-up procedure of Benjamini, Krieger, and Yekutieli post hoc test unless specified otherwise. All results are given as means ± SD. In all figures, *p < 0.05, **p < 0.01, ***p < 0.001, ****p < 0.0001.

### Calcium imaging

We also performed calcium imaging experiments with *En1-*cre mice crossed with GCaMP6f-floxp mice to express selectively the genetically encoded calcium indicator GCaMP6f into V1 interneurons. We performed a hemisection on these preparations and monitored the calcium activity from the medial aspect of the hemisected cord during drug-induced motor activity evoked by NMDA, 5-HT, and DA. The cord was placed in a recording chamber mounted on the stage of an Olympus microscope with the cut face of the hemisection facing the objective. The electrophysiological and imaging recordings started at least 30 min after the hemisection. To visualize the calcium fluorescence, the GCaMP6f protein was excited with a blue LED (415–475 nm; Excelitas), the optical signal was filtered (band-pass emission filter: 500–550), and acquisition was made with micromanager software [40] with a 20× objective (that covered a square field of view of 0.67 mm × 0.67 mm) at 16, 6, or 4 Hz (with 20-, 40-, or 50-ms exposure) using a Zyla 4.2 camera (Andor, Belfast, United Kingdom). We analyzed these signals in Matlab using the plugin MIJI (Fiji for Matlab; [41]). The video frames were first registered to minimize movement artifacts during the acquisition. We used a descriptor-based series registration with translation as a transformation model, and the registration was done against the first image only [42]. Six identical rectangular ROIs, were placed in the dorsoventral direction covering the entire field of view. In experiments in which individual neurons could be seen, we manually defined an ROI around the putative neurons of interest and measured the background calcium activity around this ROI by creating a doughnut-shaped ROI [43]. For all ROIs (rectangular, cell-shaped, and doughnut-shaped), the average pixel value was calculated in each frame, thereby creating a time series. For the cell-based ROIs, we subsequently subtracted the activity of this doughnut-shaped ROI to eliminate the contribution of signal from cells or processes lying under the cell of interest. Finally, each time series was corrected for bleaching by fitting it with a monoexponential function, which was subtracted from the data. We then calculated the minimum of each time series (f_0_) and calculated Δf = (f − f_0_) /f_0_ * 100 for each ROI. The signal was band-pass filtered at [0.05–0.8] Hz and aligned to the integrated neurograms. We also computed cycle-triggered averages of the calcium signals together with the integrated neurogram for individual cells (Fig 8B).

### Immunohistochemistry

Preparations were fixed in 4% paraformaldehyde for at least 4 h at room temperature. They were then embedded in 5% agar and sectioned transversally (60 μm) on a V1000 vibratome (Leica Biosystems, Buffalo Grove, IL, USA). The sections were then blocked in 10% normal donkey serum in 0.01 M phosphate-buffered saline (PBS) with 0.1% Triton X-100 (PBS-T) for 1.5 h and subsequently incubated overnight at room temperature in different combinations of the following antibodies: chicken anti-GFP antibody (ab13970, dilution 1:1,000, Abcam, Cambridge, MA, USA), goat anti-ChAT antibody (AB144P, dilution 1:100, Millipore, Temecula, CA, USA), rabbit anti-RFP antibody (ab62341, dilution 1:1,000, Abcam, Cambridge, MA, USA), and rabbit anti-calbindin D28k antibody (dilution 1:2,000, Swant, Marly, Switzerland). The following day, the sections were washed with PBS-T for 1 h and then incubated for 3 h with secondary antibodies: donkey anti-chicken-FITC, donkey anti-goat-Cy5, donkey anti-rabbit-Rhodamine-Red-X, donkey anti-rabbit-Dylight-405 (dilution 1:100, Jackson ImmunoResarch, West Grove, PA, USA), and DyLight 649 streptavidin (dilution 1:100, #SA-5649, Vector laboratories, Burlingame, CA, USA). The sections were then mounted on slides and coverslipped with a glycerol/PBS solution (3:7). Images were acquired using an LSM510 or LSM800 Carl Zeiss confocal microscope with 10× (air) and 40× (oil) objectives (Light imaging facility, NINDS).

## Supporting information

S1 Fig In *En1*-Arch-GFP cords, Arch-GFP expression is dense in the ventral part of the cord and surrounds motoneurons. (A–C) Z-stack projection of low-magnification (10× objective) images (5 μm per optical section) of a 60-μm transverse section of the L2 segment from a P3 *En1*-Arch mouse spinal cord showing *En1-*Arch-GFP (green, A and C), ChAT-positive (red, B and C) neurons, and the merged image (C). The white scale bars represent 100 μm. (C1) Insets from the white rectangle in the merged image show that putative motoneurons (ventrally located ChAT-positive neurons) do not express Arch (1-μm optical section). The white scale bar is 20 μm. (D–F) Z-stack projection of low-magnification (10× objective) images (5 μm per optical section) of a 60-μm section of the L5 segment from a P3, *En1*-Arch mouse spinal cord showing *En1-*Arch-GFP (green, D and F) and ChAT-positive (red, E and F) neurons and the merged image (F). The white scale bars measure 100 μm. (F1) Insets from the white rectangle in the merged image show that putative motoneurons (ventrally located ChAT-positive neurons) do not express Arch (1.05-μm optical section). The white scale bar is 20 μm. Arch, archaerhodopsin-3; ChAT, choline acetyltransferase; *En1*, *engrailed-1*; GFP, green fluorescent protein; P, postnatal day.
(TIF)

S2 Fig Dorsal root–evoked fictive locomotion in *En1*-GFP cords. (A) Fictive locomotion evoked by sacral root stimulation before (A1) and during (A2) illumination of an *En1*-GFP spinal cord. The black traces show the high-pass (10 Hz) filtered signal from the bilateral L2 and the left L5 ventral roots. The superimposed blue traces are the integrated neurograms. The green bar indicates the duration of the light. *En1*, *engrailed-1*; GFP, green fluorescent protein.
(EPS)

S3 Fig Whole-cell recordings from En1-tdT-positive neurons in dorsally shaved spinal cords during stimulation of sacro-caudal afferents. (A, B) Examples of fictive locomotion evoked by sacral dorsal root stimulation before (A1, B1) and during (A2, B2) hyperpolarization of V1 neurons in an *En1*-tdT-Arch spinal cord with the lumbar dorsal horn removed. The black traces show the high-pass (10 Hz) filtered signal from the left L2 ventral root together with intracellular recordings from *En1*-tdT-positive interneurons. The superimposed orange traces are the integrated neurograms. The green bar indicates the duration of the light. Arch, archaerhodopsin-3; *En1*, *engrailed-1*; tdT, tdTomato.
(EPS)

S4 Fig Comparison between the effect of light on locomotor-like activity *En1*-GFP and WT cords. (A) Fictive locomotion evoked by 5 μM NMDA, 10 μM 5-HT, and 50 μM DA in *En1*-GFP (A1) and WT (A2) spinal cords. The black traces show the high-pass (10 Hz) filtered signal from the right L2 and left L2 and left L5 ventral roots. The superimposed blue (A1) or gray traces (A2) are the integrated neurograms. The green bar indicates the duration of the light. (B) Time series showing the change in frequency averaged for all experiments for the bilateral flexor–dominated ventral roots. (C) Change (%) in the averaged integrated ventral root discharge (Change in MN firing) for the ipsilateral flexor (C1) and extensor (C2) ventral roots. The statistics were obtained using a bootstrap *t* test between *En1*-GFP cords (*n* = 9) and WT (*n* = 7), and the *p*-values are color coded as indicated in the box below the records. (D) Circular plots showing the phasing of the ipsilateral flexor–extensor ventral roots during fictive locomotion before (large black circles), during (large green circles), and after (large gray circles) illumination in WT (black crosses) and *En1*-GFP (blue crosses) cords. (D) R is the length of the vector, and *p* is the value for the Rayleigh test of uniformity. Using the Harrison-Kanji test, we calculated the statistical differences between the two groups of animals (genetic Identity) and the differences between the phasing in the bilateral L2 (D1) and ipsilateral L2–L5 ventral roots before, during, and after illumination (light status) (D2). There was no statistical difference in the phasing of the bilateral flexor (L2) bursts or the ipsilateral L2/L5 root bursts. The results of the test for bilateral L2 phase were light status: F(2, 47) *p* = 0.9505, genetic identity: F(1, 47) *p* = 0.5585, and interaction: F(2, 47) *p* = 0.9544. The same test was performed for the flexor–extensor phases (light status: F[2, 47] *p* = 0.8895, genetic identity: F[1, 47] *p* = 0.5286, interaction: F[2, 47] *p* = 0.8671). The data underlying this figure can be found in S8 Data. 5-HT, 5-hydroxytryptamine; DA, dopamine; *En1*, *engrailed-1*; GFP, green fluorescent protein; NMDA, N-methyl-D-aspartate; WT, wild-type.
(TIF)

S5 Fig The effect of light on drug-induced rhythmic activity in a WT hemicord. (A) Rhythmic activity evoked by 5 μM NMDA, 10 μM 5-HT, and 50 μM DA in a WT hemicord cord. The black traces show the high-pass (10 Hz) filtered signal from the left L2 and left L5 ventral roots. The superimposed gray traces are the integrated neurograms. The green bar indicates the duration of the light. 5-HT, 5-hydroxytryptamine; DA, dopamine; MN, motoneuron; NMDA, N-methyl-D-aspartate; WT, wild-type.
(EPS)

S6 Fig Comparison of the effects of light on *En1*-Arch whole and *En1*-Arch hemisected spinal cords during drug-induced rhythmic activity. (A) Time series showing the change in frequency averaged for all experiments for the ipsilateral flexor/extensor roots. (B) Change (%) in the averaged integrated ventral root discharge (Change in MN firing) for the ipsilateral extensor ventral root. The statistics were obtained using a bootstrap *t* test between *En1*-Arch cords (*n* = 16) and *En1*-Arch hemisected cords (*n* = 13), and the *p*-values are color coded as indicated in the box below the records. (C) Circular plot showing the phasing of the ipsilateral flexor–extensor ventral roots during rhythmic motor activity before (large black circles), during (large green circles), and after (large gray circles) illumination in *En1*-Arch whole (blue circles) and hemisected (yellow circles) cords. (D) R is the length of the vector, and *p* is the value for the Rayleigh test of uniformity. Using the Harrison-Kanji test, we calculated the statistical differences between the two groups of animals (cord status) and the differences between the phasing in ipsilateral L2–L5 ventral roots before, during, and after illumination (light status). The results of the test for the ipsilateral flexor–extensor phases were light status: F(2, 47) *p* = 0.035, cord status: F(1, 47) *p* < 0.0001, and interaction: F(2, 47) *p* = 0.0550. The data underlying this figure can be found in S9 Data. Arch, archaerhodopsin-3; *En1*, *engrailed-1*; MN, motoneuron.
(EPS)

S1 Data Data underlying Fig 2. (XLSX)

S2 Data Data underlying Fig 3. (XLSX)

S3 Data Data underlying Fig 4. (XLSX)

S4 Data Data underlying Fig 5. (XLSX)

S5 Data Data underlying Fig 6. (XLSX)

S6 Data Data underlying Fig 9. (XLSX)

S7 Data Data underlying Fig 10. (XLSX)

S8 Data Data underlying S4 Fig. (XLSX)

S9 Data Data underlying S6 Fig. (XLSX)

## Abbreviations

5-HT: 5-hydroxytryptamine
AMPA: α-amino-3-hydroxy-5-methyl-4-isoxazolepropionic acid
Arch: archaerhodopsin-3
CPG: central pattern generator
DA: dopamine
*En1*: *engrailed-1*
GFP: green fluorescent protein
NBQX: 2,3-dihydroxy-6-nitro-7-sulfamoyl-benzo[F]quinoxaline
NMDA: N-methyl-D-aspartate
ns: not significant
RM: repeated measures
ROI: region of interest
tdT: tdTomato
WT: wild-type
