# Supplementary figures and images for: V1 interneurons regulate the pattern and frequency of locomotor-like activity in the neonatal mouse spinal cord
Source: PLoS Biol. 2019 Sep 12;17(9):e3000447. doi: 10.1371/journal.pbio.3000447 (PMC6759197; doi:10.1371/journal.pbio.3000447)

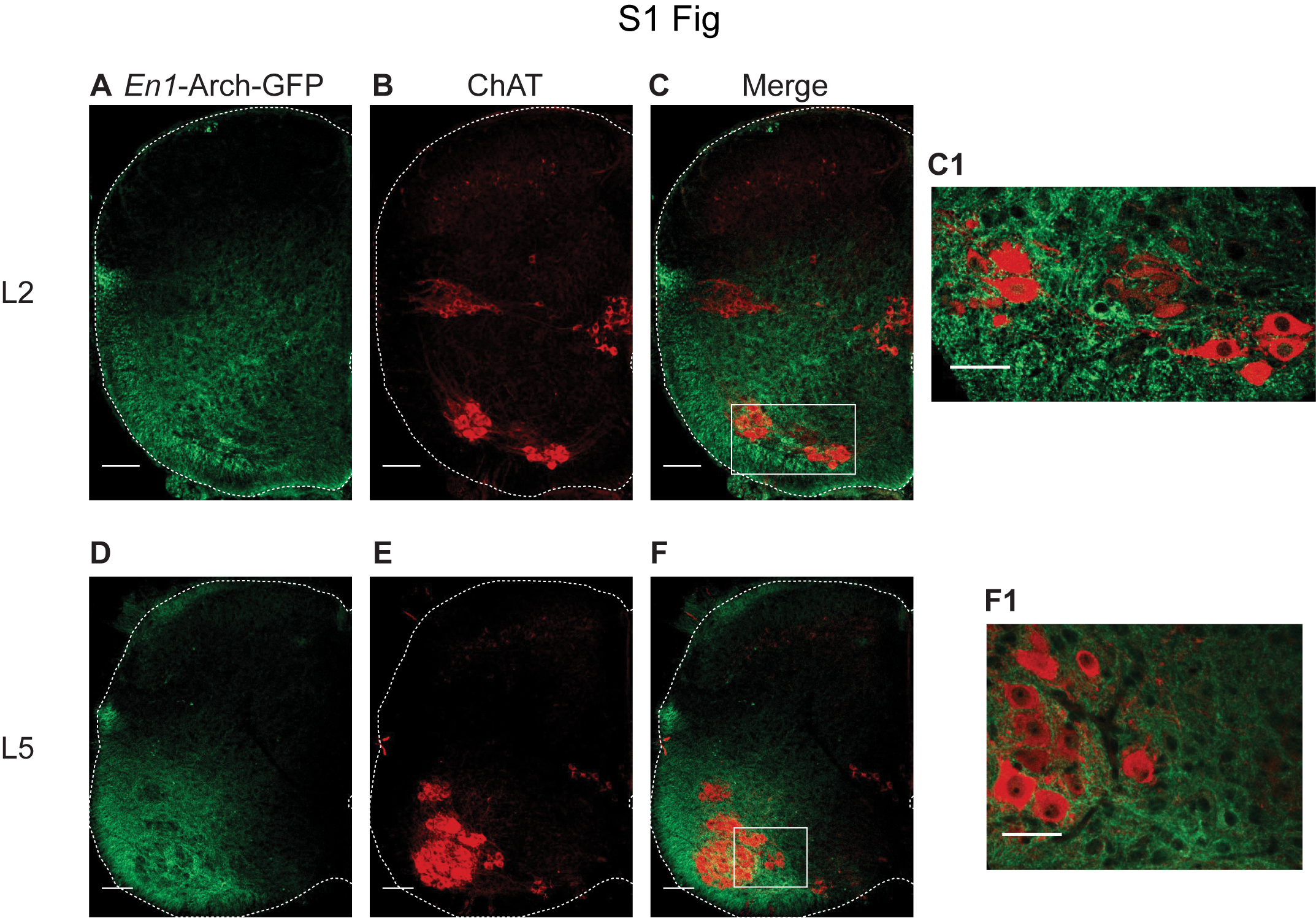

Supplement: S1 Fig — (A–C) Z-stack projection of low-magnification (10× objective) images (5 μm per optical section) of a 60-μm transverse section of the L2 segment from a P3 En1-Arch mouse spinal cord showing En1-Arch-GFP (green, A and C), ChAT-positive (red, B and C) neurons, and the merged image (C). The white scale bars represent 100 μm. (C1) Insets from the white rectangle in the merged image show that putative motoneurons (ventrally located ChAT-positive neurons) do not express Arch (1-μm optical section). The white scale bar is 20 μm. (D–F) Z-stack projection of low-magnification (10× objective) images (5 μm per optical section) of a 60-μm section of the L5 segment from a P3, En1-Arch mouse spinal cord showing En1-Arch-GFP (green, D and F) and ChAT-positive (red, E and F) neurons and the merged image (F). The white scale bars measure 100 μm. (F1) Insets from the white rectangle in the merged image show that putative motoneurons (ventrally located ChAT-positive neurons) do not express Arch (1.05-μm optical section). The white scale bar is 20 μm. Arch, archaerhodopsin-3; ChAT, choline acetyltransferase; En1, engrailed-1; GFP, green fluorescent protein; P, postnatal day. (TIF) [file pbio.3000447.s001.tif]

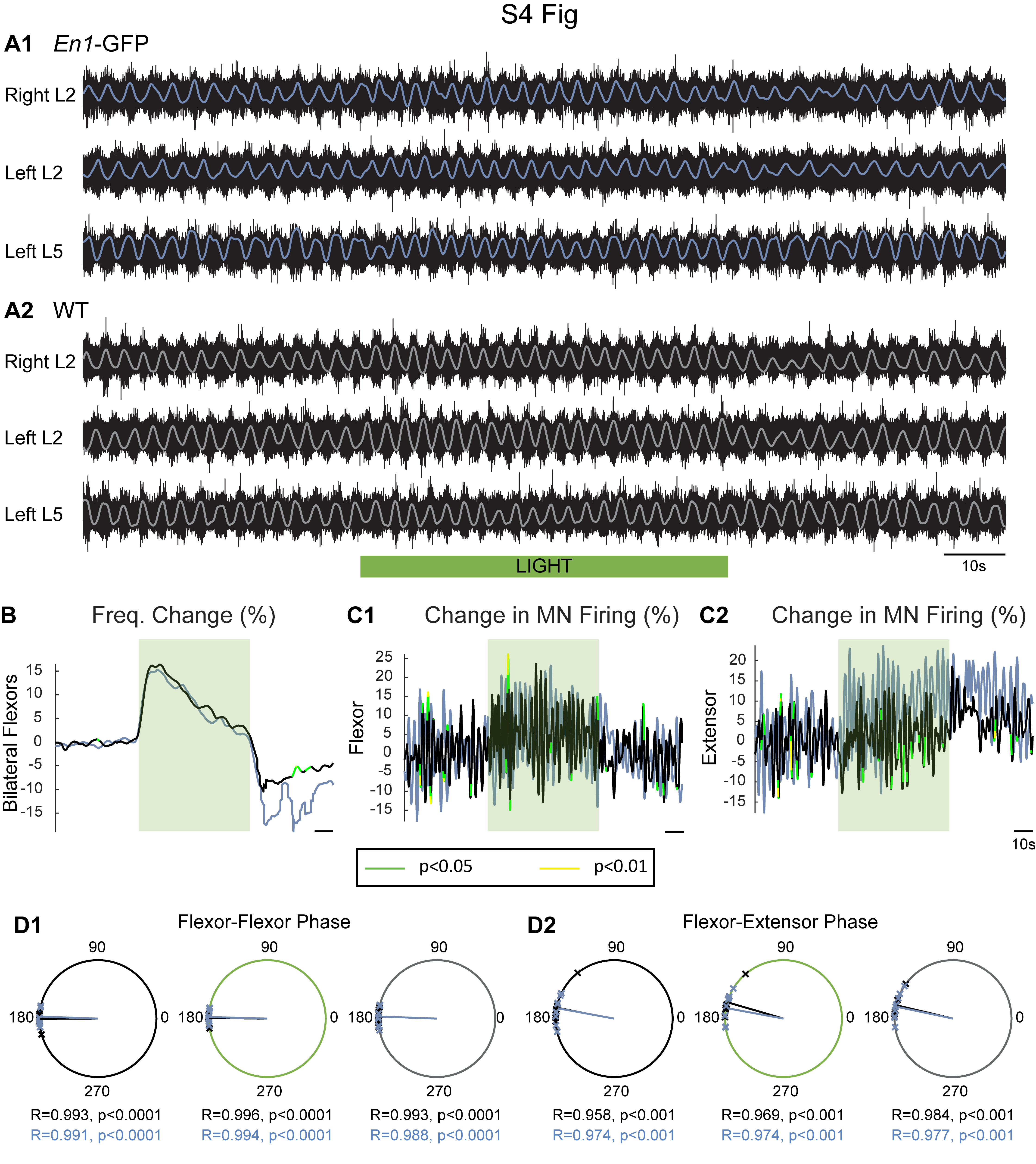

Supplement: S4 Fig — (A) Fictive locomotion evoked by 5 μM NMDA, 10 μM 5-HT, and 50 μM DA in En1-GFP (A1) and WT (A2) spinal cords. The black traces show the high-pass (10 Hz) filtered signal from the right L2 and left L2 and left L5 ventral roots. The superimposed blue (A1) or gray traces (A2) are the integrated neurograms. The green bar indicates the duration of the light. (B) Time series showing the change in frequency averaged for all experiments for the bilateral flexor–dominated ventral roots. (C) Change (%) in the averaged integrated ventral root discharge (Change in MN firing) for the ipsilateral flexor (C1) and extensor (C2) ventral roots. The statistics were obtained using a bootstrap t test between En1-GFP cords (n = 9) and WT (n = 7), and the p-values are color coded as indicated in the box below the records. (D) Circular plots showing the phasing of the ipsilateral flexor–extensor ventral roots during fictive locomotion before (large black circles), during (large green circles), and after (large gray circles) illumination in WT (black crosses) and En1-GFP (blue crosses) cords. (D) R is the length of the vector, and p is the value for the Rayleigh test of uniformity. Using the Harrison-Kanji test, we calculated the statistical differences between the two groups of animals (genetic Identity) and the differences between the phasing in the bilateral L2 (D1) and ipsilateral L2–L5 ventral roots before, during, and after illumination (light status) (D2). There was no statistical difference in the phasing of the bilateral flexor (L2) bursts or the ipsilateral L2/L5 root bursts. The results of the test for bilateral L2 phase were light status: F(2, 47) p = 0.9505, genetic identity: F(1, 47) p = 0.5585, and interaction: F(2, 47) p = 0.9544. The same test was performed for the flexor–extensor phases (light status: F[2, 47] p = 0.8895, genetic identity: F[1, 47] p = 0.5286, interaction: F[2, 47] p = 0.8671). The data underlying this figure can be found in S8 Data. 5-HT, 5-hydroxytrypta [file pbio.3000447.s004.tif]
